# Magnetic data interpretation for 2D dikes by the metaheuristic bat algorithm: sustainable development cases

**DOI:** 10.1038/s41598-022-18334-1

**Published:** 2022-08-20

**Authors:** Khalid S. Essa, Zein E. Diab

**Affiliations:** Department of Geophysics, Faculty of Science, Cairo University, P.O. 12613, Giza, Egypt

**Keywords:** Environmental sciences, Solid Earth sciences

## Abstract

Metaheuristic algorithms are increasingly being utilized as a global optimal method in the inversion and modeling of magnetic data. We proposed the Bat Algorithm Optimization (BAO) technique that is based on bat echolocation performance to find the global optimum solution. The best-estimated source parameters that correspond to the objective function minimum value are obtained after achieving the global optimum (best) solution. The suggested BAO technique does not require any prior knowledge; rather, it is a global search method that provides an effective tool for scanning the space of data to appraise sources parameters. The BAO technique is applied to magnetic data in the class of dipping and vertical dikes along 2D profiles to estimate the dimensional source parameters that include the depth to top, origin location, amplitude coefficient, index angle of magnetization, and width of the dipping dikes. The BAO technique has been used for single and multiple dikes structures. The accuracy and stability of the BAO technique are achieved on different synthetic examples of free and noisy data for single and multiple cases. Furthermore, the presented BAO technique was effectively utilized in three field examples from China and Egypt for iron ore deposits and metavolcanics basalt rock investigations. Overall, the BAO technique recovered inversion outcomes are in good agreement with borehole, geology, and published literature results.

## Introduction

Generally, magnetic survey is a powerful tool for detecting geological features and structures such as faults and dikes in geophysical exploration. Dikes structures have great economic attention and are commonly utilized as source anomalies in magnetic data interpretation^1^. Many exploration difficulties can be solved by assuming a geologic structure that is connected to dikes (i.e., dipping and vertical). These dikes models are usually used in magnetic interpretation to get the depth, location, and width of a group of geologic structures^2^. Recognition of these basic geometries of dikes provides general insights into the origins of other important ore deposits. Therefore, their study is important due understanding the tectonic setting, volcanic intrusion formation, in addition to their economic mineralogical significance^3^. The magnetic survey is conducted to investigate the geological structure of the underlying through the anomalies of the Earth's magnetic field caused by the magnetic minerals that have been contained in the subterranean rocks^4^. The survey involves mapping one or more components of the earth's geomagnetic field in order to investigate magnetic abnormalities or anomalies. The magnetic anomalies are interpreted by pointing out the source buried bodies' spatial position, depth, and magnetic characteristics (magnetic susceptibility).

Recently magnetic surveys play an essential role in geothermal investigation^5^, environmental and engineering applications^6^, geotechnical engineering^7^, archaeological exploration^8,9^, hydrological investigation^10^, unexploded military ordnance (UXO) mapping^11^, and geotectonic studies^12^. Furthermore, magnetic methods have a wide range of applications in visualizing and mapping economic ore deposits ^13–19^. Magnetic surveys have been employed in a variety of applications, including oil and gas exploration^20^, dikes location^17,21–23^, buried metallic-bodies^24^, cavity detection^25^, landfill investigation^26^, depth estimation to the basement^27^, and intrusions of plutonic igneous rocks^28^.

Magnetic anomalies are inferred utilizing simple geometric models (point sources, dikes, spheres, horizontal and vertical cylinders, and prisms) to estimate model parameters. Several graphical and numerical approaches for analyzing magnetic data have been created using simple geometric models, for example, the matching curve, nomograms, and characteristic points methods are examples of these approaches^29–32^, Werner and Euler deconvolution^33–35^, moving average techniques^36^, least-squares approaches^36,37^, Fourier transforms^38,39^, alternative local wave number technique^40^, numerical gradient-based technique^17^, tilt-angle methods^41,42^, correlation techniques^43^, and spectral analysis techniques^44^. However, most of these methods have several defects such as individual subjectivity, use of only a few data points along with the measurement profile, hypersensitivity to noise, and influence of adjacent effect (which might degrade the accuracy of the results). Moreover, they require initial model parameters depending upon the geological data, which the final solutions often get trapped in local minima than global minima^45^ and depended on a priori knowledge, which is not always accessible^18^.

On the other side, metaheuristic algorithms were developed to interpret the geomagnetic data, which rely on searching for global optimum solution that is more accurate and efficient than graphical and numerical methods^46^. Metaheuristic algorithms such as simulated annealing technique (SA)^47,48^, genetic algorithm (GA)^49^, particle swarm optimization (PSO)^50,51^, neural networks approach (NN)^22,52^, differential evolution algorithm (DE)^53^, and ant-colony optimization algorithm (ACO)^54^. These algorithms are popular among researchers because they are more adaptable and capable of dealing with a wide range of problems than traditional optimization techniques. The proposed Bat algorithm optimization (BAO) approach in the present study lies in the metaheuristic algorithms categories and represents a novel technique for interpreting the geomagnetic data (i.e., dikes). The BAO approach is an efficient optimization approach for resolving complex problems rapidly, consistently, and precisely.

In addition, the proposed BAO approach has several advantages: (1) it may provide highly quick convergence by switching from exploration to exploitation at a reasonably early stage, (2) it may be used as both a global and a local optimizer, (3) it is capable to handling multi-model problems effectively, and (4) As the iteration advances, BAO uses the controlling parameter to update the parameter and is committed to preserving the population's diversity of solutions. Due to these conspicuous superiority of BAO, BAO has been improved and used in a variety of fields, including the optimal independent micro-smart grid, the economic scheduling problem, fault diagnosis on low-speed rolling bearing, multi-objective function optimization, and the optimization of echo state networks^55–59^. In contrast, the defects of the BAO approach lie in the following items: (1) It has a lack of good exploration, (2) It requires the parameter tuning to achieve better search output, and (3) The switching between exploration and exploitation requires a better control strategy. Accordingly, certain study findings indicate that when problem dimensions rise, the system's performance may suffer and its ability for exploration may deteriorate. Numerous academics have investigated and used this approach, as well as developed comparable enhancement ideas, to address this flaw^60–65^.

In the current study, we applied a BAO approach to interpreting magnetic data transmitted across 2D profiles by a certain basic geometrical shapes as dipping and vertical dikes models and multiple-source models. The goal of this research is to invert the magnetic data to calculate the model parameters of the causative buried body, which are depth (z), source origin location (x_o_), amplitude coefficient (K), index parameter angle (θ), and dike width (d). As the program reaches the global best solution, the preferred interpretive model parameters are achieved to correspond to the minimum of normal root-mean-square error (NRMSE) of the objective function. The proposed BAO approach is applied to various numerical examples of simple geometrical shapes (dipping dikes, vertical dikes, or thin sheets), multiple-source models, and various field examples for ore deposits and metavolcanics basalt rock investigation.

The following is the structure of the paper: The principles of echolocation are covered in Sect. 2, as the traditional formulation of the bat algorithm. Section 3 covers the forward modeling and formulation of the proposed BAO approach, while Sect. 4 describes how to invert magnetic data using the BAO approach. Section 5 supplies that the suggested BAO approach is confirmed and verified by applying numerical examples (including free and noisy examples for different single models and investigating the interference of multiple models with purely and contaminated data). Section 6 shows and discusses the applicability of the given BAO approach to various real field examples from different areas. Finally, Sect. 7 highlights conclusions, which summarizes the objectives of the present BAO approach.

### Bat algorithm optimization (BAO)

The Bat Algorithm Optimization (BAO) is a nature-inspired metaheuristic optimization technique and was first introduced mathematically by Yang^66^. It depends on the echolocation characteristic of microbats. Microbats use echolocation in the dark to identify their nest, avoid obstacles, and track prey. Bats produce a loud sound pulse in the range of 8–10 kHz and listen for echoes from nearby objects. Each pulse lasts only a few milliseconds (up to about 8 to 10 ms). When bats are approaching prey or an item, their pulse rate increases, but their sound loudness falls^66^. So, the echolocation activity of microbats may be represented in a way that maximizes or optimize objective functions. In brief, the key rules of the global optimizing bat algorithm are: (1) Bats use echolocation to determine distance; (2) Bats detect their sources by flying at a specified frequency range [Q_min_, Q_max_] with an initial velocity of (*V*_*i*_) at position (*X*_*i*_); and (3) the loudness (*L*_*i*_) and the pulse emission rate, (*r*_*i*_), which vary relied on the space or distance amongst the target object and the bat.

**Figure 1:**
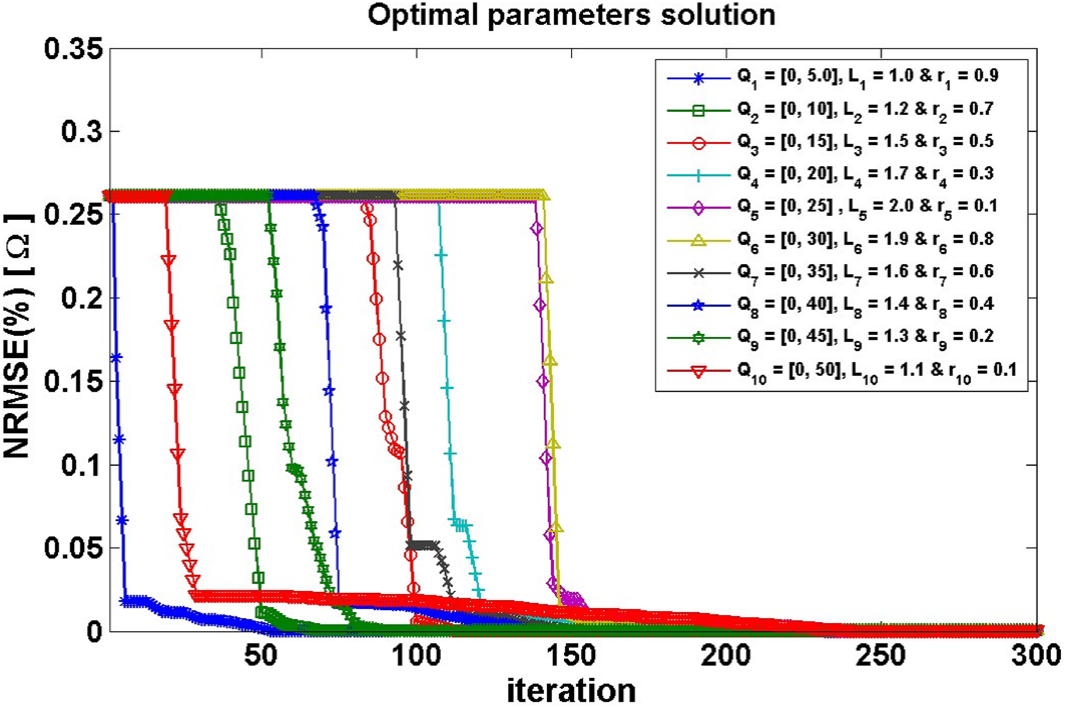
The effect of different sets of optimization parameters (*Q*_*i*_, *L*_*i*_ and *r*_*i*_) on the convergence rate of the BAO approach technique.

The frequency range [Q_min_, Q_max_] is referred to by the wavelength spectrum [K_min_, K_max_]. As a result, in an optimization problem, changing the frequency or wavelength may be utilized to vary the movement range of bats (Eqs. 1–3). As a result, selecting the appropriate frequency or wavelength range is critical, and it should be selected to fit the scale of the interest region before toning down to lower ranges. The spectrum of [0, 5] was calculated as the optimal frequency range in this inquiry after executing the technique with varied settings (Fig. 1). The pulse rate, r_i_, can range from 0 to 1, with 0 denoting no pulses and 1 denoting the highest pulse emission rate. Furthermore, the initial loudness, i.e., L_i_, might often be in the ^1,2^ range^66^. As the bats get closer to their target, their loudness drops but their pulse emission rate rises. The algorithm updates the rate of emission and the loudness of the bats when a new solution is improved, implying that the bats are reaching the best solution (Eqs. 4–5)^67^.

The effects of the optimizing parameters of the frequency (*Q*_*i*_), Loudness (*L*_*i*_), and rate of pulse emission (*r*_*i*_) on the rate of BAO approach convergence were studied (Fig. 1) using different ranges of each parameter. The influence of each set of (*Q*_*i*_, *L*_*i*_, and *r*_*i*_) parameters on the convergence rate and behavior is shown in Fig. 1. Figure 1 suggests that the optimum set has Q_1_ = [0, 5], L_1_ = 1.0 and r_1_ = 0.9, which has a minimum NRMSE of the objective function than other sets and gives a fast convergence to the optimum solution.

The performance of the BAO code to obtain the optimal model parameters of the assumed model (i.e. Numerical model-1) have been measured using the MATLAB function “tic & toc” to measure the wall-clock time, it takes about 41 s on simple PC. This result indicates that the fastest performing of BAO algorithm compared with the other metaheuristic algorithms such as particle swarm optimization (PSO), cuckoo search algorithm (CS), and artificial bee colony algorithm (ABC)^68^. This characteristic of BAO can be attributed to the parameter tuning features.

The following are the equations that show the link between algorithm parameters^66^:

1 $$Q_{i}^{t} = Q_{min} + \left( {Q_{max} - Q_{min} } \right)\beta$$

2 $$V_{i}^{{\left( {t + 1} \right)}} = V_{i}^{t} + \left( {X_{i}^{t} - X_{best} } \right)Q_{i}^{t}$$

3 $$X_{i}^{{\left( {t + 1} \right)}} = X_{i}^{t} + V_{i}^{{\left( {t + 1} \right)}}$$

4 $$L_{i}^{{\left( {t + 1} \right)}} = \alpha L_{i}^{t}$$

5 $$r_{i}^{t} = r_{i}^{0} \left[ {1 - {\text{exp}}\left( { - \gamma \tau } \right)} \right]$$

where, *Q*_*i*_ represents the spectrum frequency of *i*^*th*^ bat which is updated in every iteration process, $\beta$ represents a uniformly random vector in the range [0, 1] and X_best_ represent the current global best solution through all numbers of the bats, $\alpha$ and $\gamma$ are constants, 0 < $\alpha$  < 1 and $\gamma$ > 0 and $\tau$ is the scaling factor.

The BAO approach utilizes a random path to produce new results from every chosen best solution in the local search, as follows:

6 $$X_{new} = X_{old} + \varepsilon A^{t}$$

where $\varepsilon$ $\in$ [–1, 1] represents a random number, and *L*^*t*^ is the average loudness of all number of the bats at the current process.

Generally, in respect of accuracy and performance, the BAO approach outperforms most other algorithms. The global optimizing bat algorithm becomes a normal PSO when the frequency perturbations are replaced with a random parameter when L_i_ = 1 and r_i_ = 1 are set. Similarly, by substituting the velocities with constant loudness and pulse rate, the BAO approach is reduced to a basic harmony search algorithm.

## Methodology

### Magnetic forward modeling

**Figure 2:**
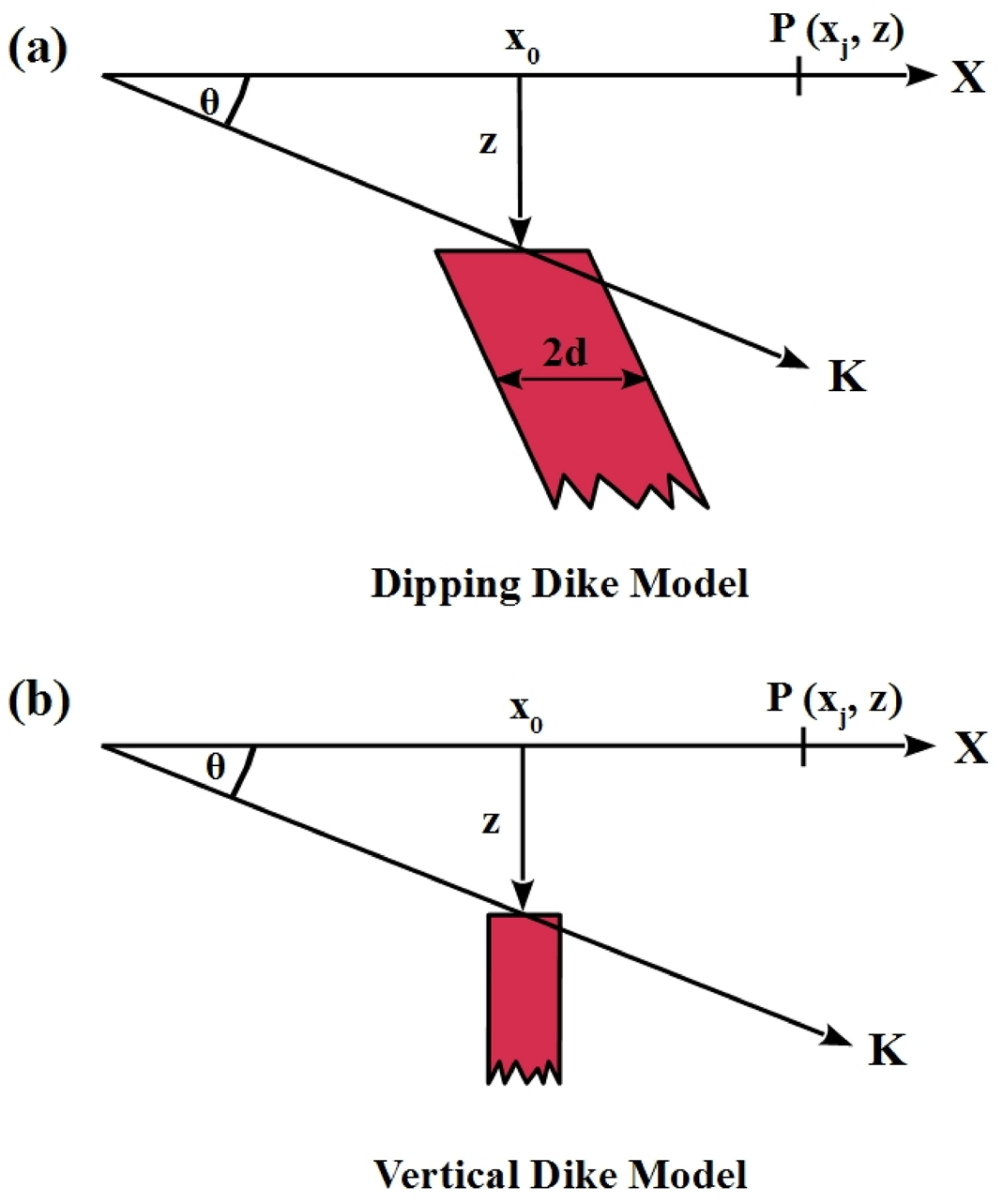
Geometrical shaped model configurations: (**a**) Dipping dike model, and (**b**) Vertical dike model (thin sheet model).

The total magnetic anomaly effect (T) at a stationary point (x_j_) along profile due to a 2D dipping dike of infinite strike length, semi-infinite depth extension, and uniformly magnetization (Fig. 2a) is provided by^17,30,69^:

7 $$T\left( {x_{j} ,x_{o} , z, \theta , d, K} \right) = K\left[ {\sin \theta \left( {\tan^{ - 1} \left( {\frac{{(x_{j} - x_{o} ) + d}}{z}} \right) - \tan^{ - 1} \left( {\frac{{(x_{j} - x_{o} ) - d}}{z}} \right)} \right) - \frac{\cos \theta }{2}\ln \left( {\frac{{((x_{j} - x_{o} ) + d)^{2} + z^{2} }}{{((x_{j} - x_{o} ) - d)^{2} + z^{2} }}} \right)} \right], j = 1,2,3, \ldots ,n$$

where *x*_*j*_ and *x*_*o*_ represent the profile distance (m) of the stationary points and the origin of the covered source (Fig. 2a), *z* is the depth to the top of the dipping dike (m) (Fig. 2a), *θ* is the index parameter angle (degrees), d denotes the half width of the dipping dike (m), *K* is the amplitude coefficient (nT), and *n* represents the data point numbers along with the profile.

The general formula for a magnetic anomaly (T) due to thin vertical dike (total, horizontal or vertical fields) (Fig. 2b) is provided by^29,70,71^:

8 $$T\left( {x_{j} ,x_{o} , z, \theta ,K} \right) = K\left[ {\frac{{z \cos \theta + \left( {x_{j} - x_{o} } \right)sin \theta }}{{ \left( {x_{j} - x_{o} } \right)^{2} + z^{2} }}} \right], j = 1,2,3, \ldots , n$$

where *x*_*j*_ and *x*_*o*_ are the stations along the profile and the origin of the hidden source structure (m) (Fig. 2b), *z* represents the depth to the top of the vertical thin dike (m) (Fig. 2b), *θ* is the index parameter angle (degrees), *K* is the amplitude coefficient (or effective magnetization intensity) (nT), and *n* represents the data point numbers.

### Inversion process

It's critical to have precise findings for the subsurface model parameters while assessing magnetic data to match the observed data. As a result, a high-capacity inversion technique was required to accurately estimate subsurface model parameters (depth, position, and form of the buried anomalous body, among other things). Metaheuristic inversion techniques are beneficial in several case studies. In comparison to metaheuristic inversion algorithms, traditional inversion approaches are more complex, time-consuming, and inefficient.

In this work, we propose an approach to invert magnetic data based on Yang (2010). The depth (z), location (x_o_), index angle (θ), half width (d), and amplitude coefficient (K), are the primary essential characteristics factors that describe the magnetic data anomaly due to dike model. As a result, these factors are investigated in the proposed BAO inversion method to establish a subsurface model that matches the real ones.

The placement of each bat in the search space implies a solution. Bats fly randomly in search space and apply a solution in each iteration step. The point with the lowest misfit of the NRMSE of the objective function determines the ideal best solution (X_best_). This technique is carried out a certain number of times. The X_best_ is picked as the best response after the final iteration. In this study, the BAO inversion program was established and tested on various numerical cases and real-world datasets.

The process steps that make up the proposed BAO methodology for inverting magnetic data consist of the following:

1. The virtual bats' initial position *X*_*i*_ (*i* = 1, 2, 3,…, N), frequency *Q*_*i*_, velocity *V*_*i*_, loudness *L*_*i*_, and pulse rates of emission *r*_*i*_ are as follows: In the search space, each bat characterizes a potential solution. The distinctive source parameters (i.e,. z, x_o_, d, and K) are randomly chosen from the search space to represent the variable *X*_*i*_, while *V*_*i*_ represents the velocity for every unique virtual bat.
2. Determining the X_best_:
  The objective function (Ω) is defined as the NRMSE between measured and calculated magnetic data anomalies and is also known as the misfit function. It is a crucial component of any optimization and varies based on the issue type. For the interpretation of magnetic data confined to basic geometric shapes (e.g., dipping dike and vertical thin sheet models), the following misfit objective function (Ω) between observed and model response has been applied:
  9 $${\Omega } = 100\sqrt {\frac{1}{N}\mathop \sum \limits_{j = 1}^{N} \left[ {\frac{{T_{Obs} - T_{Cal} }}{{\left| {T_{Obs} } \right| - \max (T_{Obs} ) - \min \left( {T_{Obs} } \right) }}} \right]^{2} ,}$$
  where *N* is the number of data points, *T*_*Obs*_ denotes the magnetic data observed, and *T*_*Cal*_ denotes the calculated magnetic model response. The forward modeling approach may be used to compute *T*_*Obs*_. The misfits are first evaluated using Eq. (9), and the bat with the lowest misfit is picked as the X_best_.
3. Run the following instructions while the program is still iterating for the maximum amount of iterations:
  To create a new solution, adjust the frequency spectrum (Eq. 1), update the velocities, and position/solutions (Eqs. 2 and 3).
  if *rand* > *r*_*i*_.
    choose a solution amongst the best solution.
    build a local solution around the chosen best solution (Eq. 6).
  end if
  if *rand* < *L*_*i*_ and *Ω* (*X*_*i*_) < *Ω* (X_best_).
    accept the new solutions.
    increase *r*_*i*_ and reduce *L*_*i*_ (Eqs. 4 and 5).
  end if
  Rank the bats and find the current X_best_
  end while

**Figure 3:**
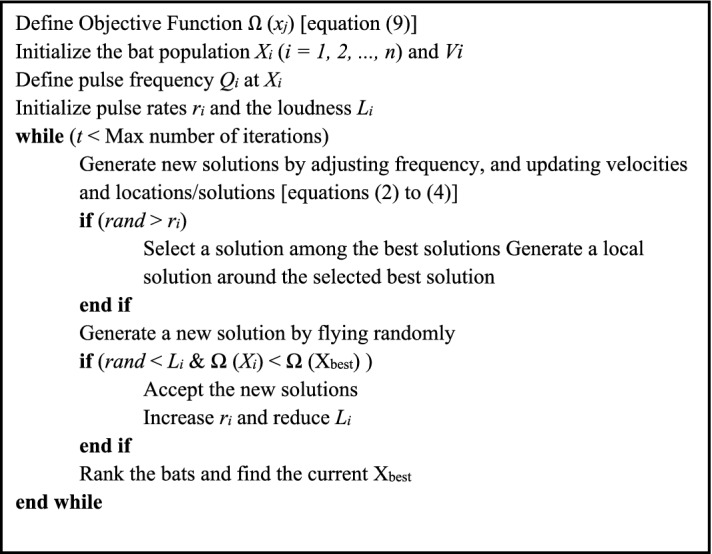
Pseudo code of the Bat algorithm optimization (BAO) (modified after Yang, 2010).

**Figure 4:**
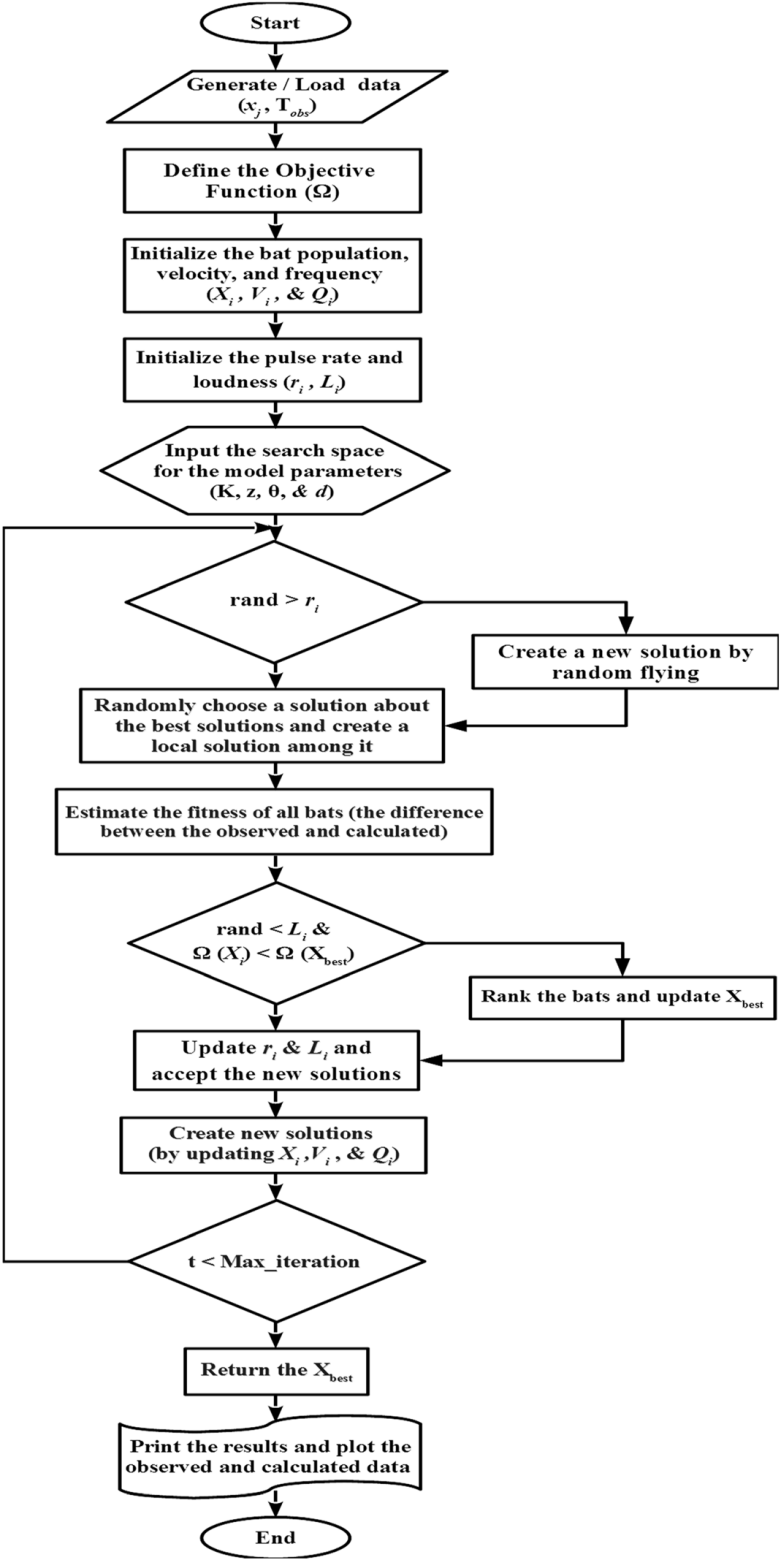
Flowchart shown the essential elements of BAO approach.

The basic features of the BAO technique are explained in the pseudocode displayed in Fig. 3 and the flow chart shown in Fig. 4.

### Numerical dataset examples

The effectiveness and validity evaluation of the proposed BAO technique in recognizing and inverting magnetic data were tested on various numerical dataset examples. The numerical examples are based on the categories of geometrical forms (2D dipping dike, vertical dike, or thin sheet models). Also, the stability and accuracy of the proposed technique are examined on noisy data and explored the interference influence of neighbour structures.

### Numerical Model-1

**Figure 5:**
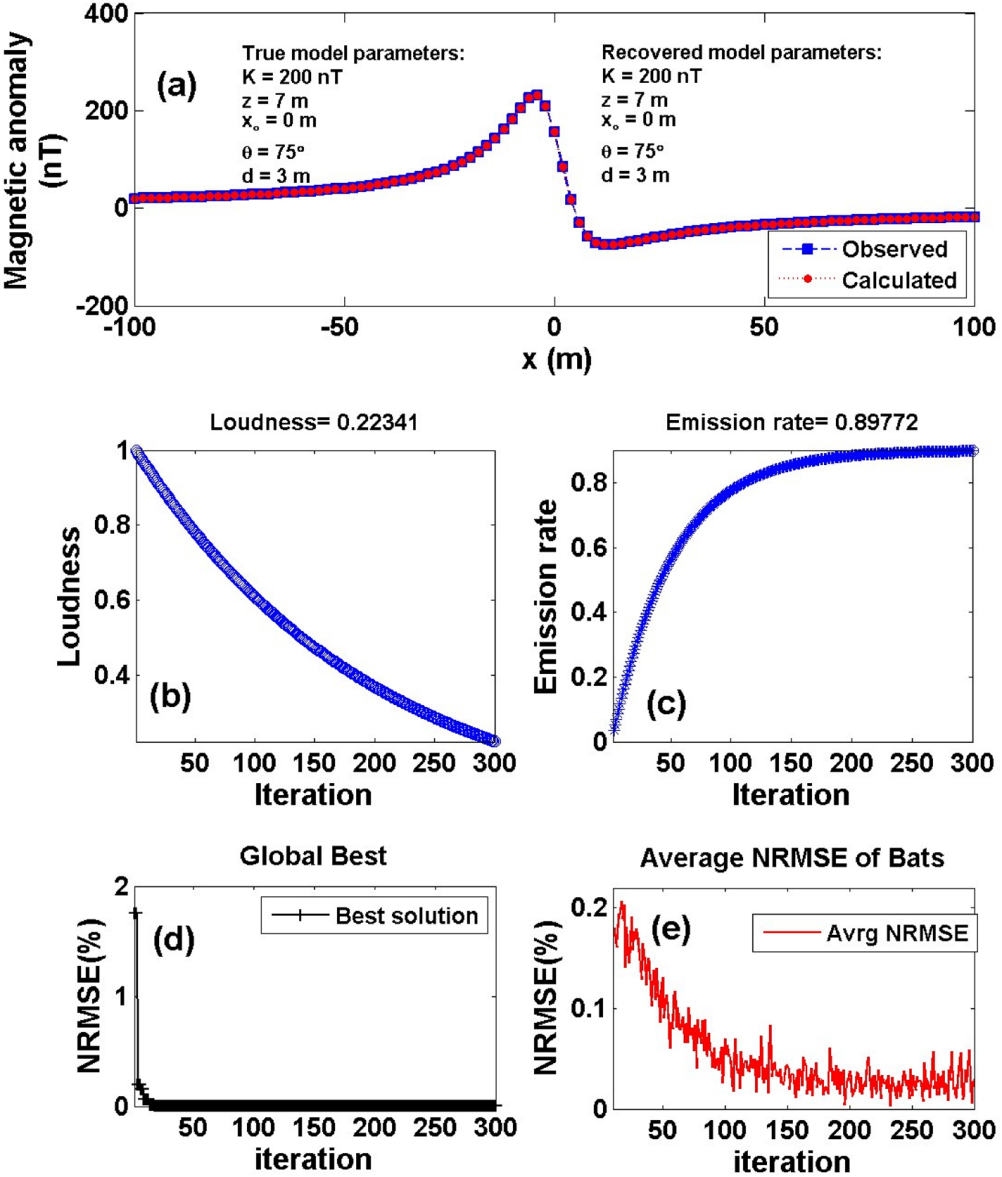
Numerical Model-1: noise-free numerical example of the 2D dipping dike model. (**a**) The measured magnetic anomaly generated by 2D dipping dike model (True model parameters), as well as the calculated magnetic anomaly (Recovered model parameters) using BAO approach, (**b**) loudness of the bats, (**c**) emission rat of the bats, (**d**) NRMSE of the global best solution (Ω) of the bats versus the iteration numbers, and (**e**) the average NRMSE of all the bats.

First, a noise-free numerical example of a 2D dipping dike model has been explored (i.e. model a) as illustrated in Fig. 2. Equation (7) is used to calculate the magnetic response of the 2D dipping dike model with *K* = 200 nT, *z* = 7 m, *x*_*o*_ = 0 m, *θ* = 75 °, and *d* = 3 m, and a profile length of 201 m (Fig. 5a). The measured data that has to be evaluated is represented by this anomalous response. Following the processes outlined in Sect. 2 of the BAO approach, the average bat loudness vs. iteration numbers is shown in Fig. 5b.

The average bat loudness vs. iteration numbers is shown in Fig. 5b. The number of iterations is determined by estimating the objective function's minimum NRMSE and achieving the optimal magnetic anomaly response solution (Fig. 5a). Figure 5c depicts the emission rat created by the bats throughout each iteration step, showing that as the bats come closer to their goal, their loudness lowers but their pulse emission rate rises. Figure 5d displays the NRMSE of the global best solution (i.e., min objective function, Ω) vs the iteration numbers, which demonstrates that after 300 iterations, it approaches the min for all bat numbers. For each iteration phase, Fig. 5e displays the average NRMSE of all the bats.

The global optimal solution of the magnetic anomaly response (i.e., the model parameters of the model) is achieved when the objective function (*Ω*) approaches the minimum of the NRMSE during the iteration operation. Table 1 provides that the recovered model parameters of the noise free numerical example are equal to the actual model parameters when the objective functions (Ω) approach the minimum. This means that the BAO technique used is accurate, stable, and capable of recovering the real values of the model parameters. In addition, Table 1 shows search space and relative errors (RE) for each model parameter.

**Table 1:** Numerical Model-1: True and recovered model parameters of the noise-free numerical example and the corresponding RE.

Model parameters	True value	Search range	Recovered value	RE (%)	*Ω*
*K* (nT)	200	100:500	200 ± 0.00	0.00	0.000000
*z* (m)	7	1:10	7 ± 0.00	0.00	
*x*_*o*_ (m)	0	– 100:100	0 ± 0.00	0.00	
*θ* (^o^)	75	5:90	75 ± 0.00	0.00	
*d* (m)	3	1:5	3 ± 0.00	0.00	

**Figure 6:**
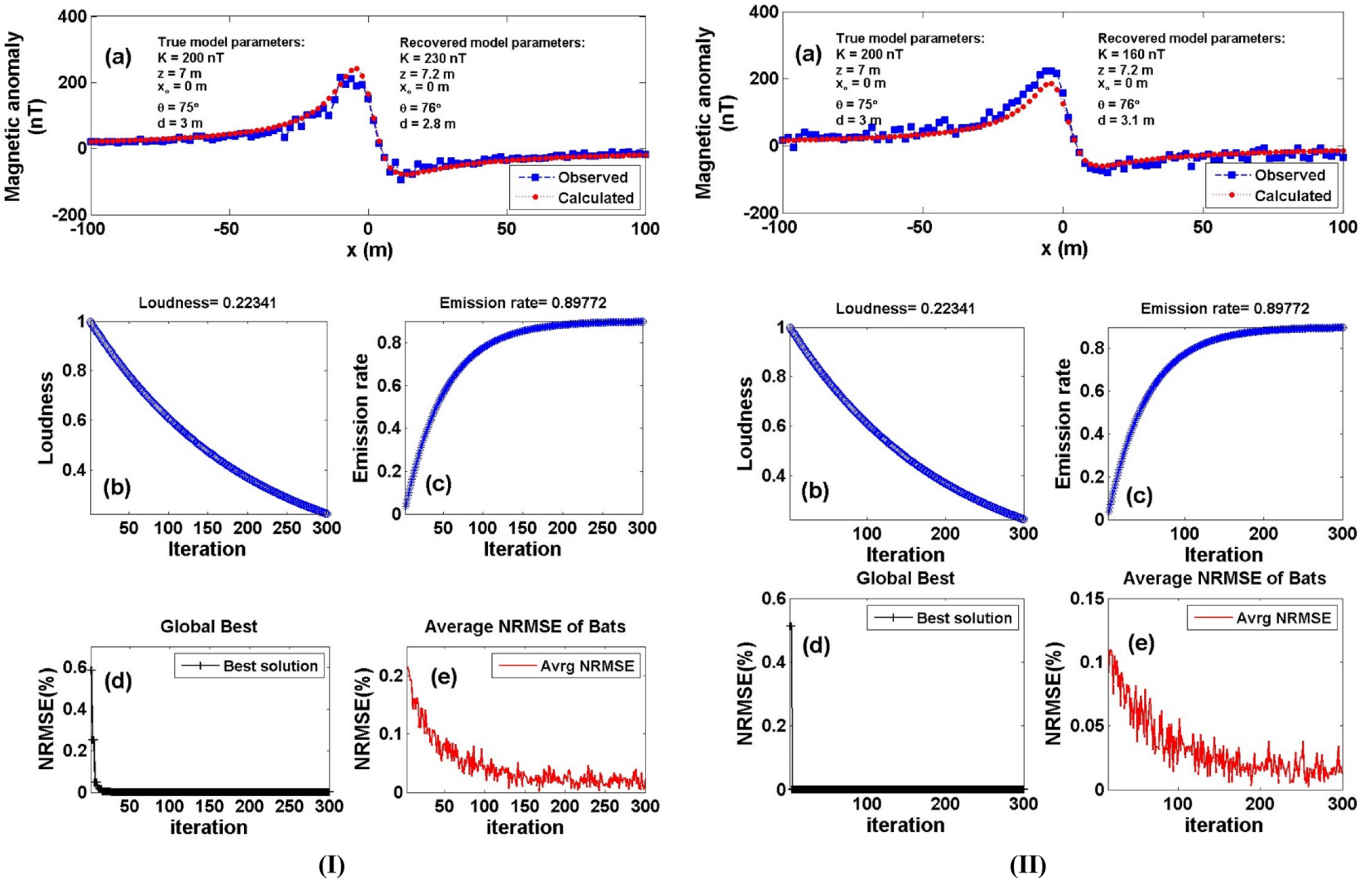
Numerical Model-1: noisy numerical example of the 2D dipping dike model (Fig. 5a) after contaminated with 15% RGN (I), and 15% AWGN (II). (**a**) Noisy magnetic anomaly generated by 2D dipping dike model (True model parameters) + noise, as well as the calculated magnetic anomaly (Recovered model parameters) using BAO approach, (**b**) loudness of the bats, (**c**) emission rat of the bats, (**d**) NRMSE of the global best solution (Ω) of the bats versus the iteration numbers, and (**e**) the average NRMSE of all the bats.

To check the stability of the proposed BAO approach, we have introduced two different kinds of noise, the random Gaussian noise (RGN) and the additive white Gaussian noise (AWGN) to the noise-free data presented in Fig. 5a using a noise percentage of 15%. By applying the aforementioned procedures of the BAO approach to the noisy data anomalies, the best obtained model parameter of the recovered model will be corresponding to the minimum NRMSE of the objective functions (*Ω*). Figure 6 shows the noisy contaminated magnetic anomaly after adding the 15% RGN (I) and 15% AWGN (II) to the data presented in Fig. 5a, respectively as the calculated magnetic response after obtaining the best model parameters using the BAO inversion approach (Panel a). The loudness, emission rate, the NRMSE of the global best solution (*Ω*), and the average NRMSE of all the bats are shown in panels (b), (c), (d), and (e) of Fig. 6, respectively.

Table 2 shows the corresponding recovered model parameters of the noisy numerical examples for introduced noise types (RGN and AWGN). The obtained results show that the recovered model parameters are not significantly affected by the intruding noise and are close to the true ones. Also, Table 2 illustrates the RE corresponding to each obtained model parameter for each kind of noise (RGN and AWGN). It can be concluded that the BAO approach proposed here is stable to the two introduced types of noises.

**Table 2:** Numerical Model-1: True and recovered model parameters of the 15% noisy numerical example and the corresponding RE.

Model parameters	True value	Search range	Recovered value		RE (%)		*Ω*	
			15% RGN	15% AWGN	15% RGN	15% AWGN	15% RGN	15% AWGN
*K* (nT)	200	100:500	230 ± 28.72	160 ± 29.04	15.00	20.00	1.06*10^–6^	1.7*10^–6^
*z* (m)	7	1:10	7.2 ± 0.60	7.2 ± 0.60	2.86	2.86		
*x*_*o*_ (m)	0	– 100:100	0 ± 0.00	0 ± 0.00	0.00	0.00		
*θ* (^o^)	75	5:90	76 ± 3.34	76 ± 3.35	1.33	1.33		
*d* (m)	3	1:5	2.8 ± 0.28	3.1 ± 0.27	6.67	3.33		

To further check the stability of the proposed method with respect to the amount of noises, we have increased the percentage of noise to 20% for both the kind of noises (RGN & AWGN) and reprocess the same procedure of the BAO approach to the synthetic noisy anomalies. Table 3 shows the recovered model parameters for the 20% noisy numerical examples for both kinds of noise (RGN and AWGN). The obtained results show that the recovered parameters are still having good results in the presence of the 20% of noise for the both kinds (RGN and AWGN). Also, Table 3 illustrates the RE corresponding to each obtained model parameter for each kind of noise in case of 20% noise amount. Finally, it can be concluded that the BAO approach anticipated here is stable with respect to noise types and amounts for 2D dipping dike model case.

**Table 3:** Numerical Model-1: True and recovered model parameters of the 20% noisy numerical example and the corresponding RE.

Model parameters	True value	Search range	Recovered value		RE (%)		*Ω*	
			20% RGN	20% AWGN	20% RGN	20% AWGN	20% RGN	20% AWGN
*K* (nT)	200	100:500	150 ± 33.54	140 ± 42.50	25.00	30.00	3.15*10^–6^	1.05*10^–5^
*z* (m)	7	1:10	7.5 ± 0.70	7.8 ± 0.59	7.14	11.43		
*x*_*o*_ (m)	0	– 100:100	2 ± 2.67	1 ± 2.06	0.00	0.00		
*θ* (^o^)	75	5:90	70 ± 3.35	78 ± 3.26	6.67	4.00		
*d* (m)	3	1:5	2.8 ± 0.33	3.4 ± 0.33	6.67	13.33		

### Numerical Model-2

**Figure 7:**
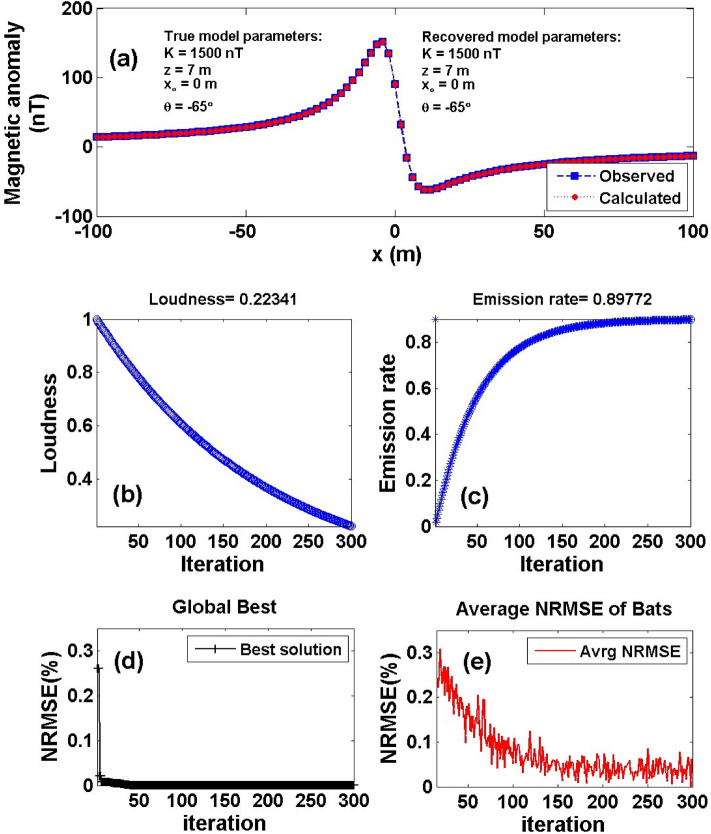
Numerical Model-2: noise-free numerical example of the vertical dike (or thin sheet) model. (**a**) The measured magnetic anomaly generated by vertical dike (or thin sheet) model (True model parameters), as well as the calculated magnetic anomaly (Recovered model parameters) using BAO approach, (**b**) loudness of the bats, (**c**) emission rat of the bats, (**d**) NRMSE of the global best solution (Ω) of the bats versus the iteration numbers, and (**e**) the average NRMSE of all the bats.

To further investigate the BAO algorithm for studying the geological structures, a noise-free example of a vertical dike (or thin sheet) model has been investigated (model b) (Fig. 2). The magnetic anomaly of the vertical dike model *K* = 1500 nT, *z* = 7 m, *x*_*o*_ = 0 m and *θ* = -65 ° is calculated using Eq. (8) for a 201-m long profile (Fig. 7a). Applying the same procedure of the BAO algorithm described above. The computed magnetic response of the vertical dike model is shown in Fig. 7a. The average loudness of the magnetic anomaly is shown in Fig. 7b, and the emission rate of the bat is obtained in Fig. 7c. The NRMSE of the global best solution (min objective function, *Ω*) is shown in Fig. 7d, and the average NRMSE of all the bats is shown in Fig. 7e. Table 4 shows that the recovered model parameters of the vertical dike model are extremely identical to the actual one. The result supports that the BAO algorithm can be used to investigate the geological structure, like vertical dikes or thin sheets. In addition, Table 4 illustrates the RE of each recovered model parameter.

**Table 4:** Numerical Model-2: True and recovered model parameters of the noise-free numerical example and the corresponding RE.

Model parameters	True value	Search range	Recovered value	RE (%)	*Ω*
*K* (nT)	1500	500:2500	1500 ± 0.00	0.00	0.000000
*z* (m)	7	1:10	7 ± 0.00	0.00	
*x*_*o*_ (m)	0	–100:100	0 ± 0.00	0.00	
*θ* (^o^)	65	– 5:– 90	– 65 ± 0.00	0.00	

**Figure 8:**
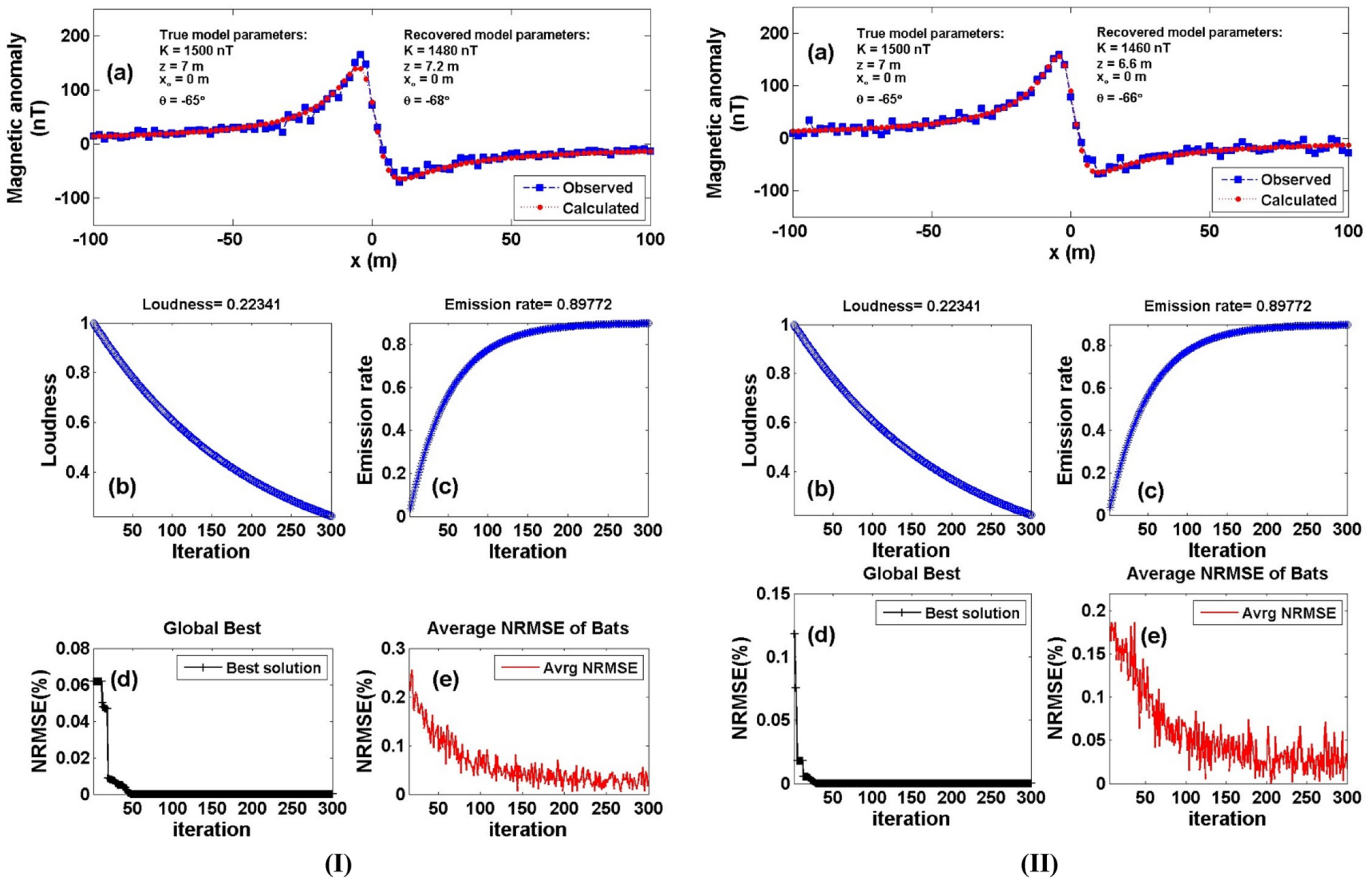
Numerical Model-2: noisy numerical example of the vertical dike (or thin sheet) model (Fig. 7a) after contaminated with 15% RGN (I), and 15% AWGN (II). (**a**) Noisy magnetic anomaly generated by the vertical dike (or thin sheet) (True model parameters) + noise, as well as the calculated magnetic anomaly (Recovered model parameters) using BAO approach, (**b**) loudness of the bats, (**c**) emission rat of the bats, (**d**) NRMSE of the global best solution (Ω) of the bats versus the iteration numbers, and (**e**) the average NRMSE of all the bats.

Moreover, Fig. 7a is contaminated with the two different kinds of noises mentioned above RGN and AWGN (Fig. 8a). The best model parameter of the recovered model will be corresponding to the minimum NRMSE of the objective functions (*Ω*). Figure 8 shows the contaminated magnetic anomaly response and the calculated magnetic response after obtaining the best model parameters using the BAO inversion approach (Panel a). The loudness, emission rate, the NRMSE of the global best solution (*Ω*), and the average NRMSE of all the bats are shown in panels b, c, d, and e of Fig. 8, respectively. Table 5 shows that the recovered model parameters of the contaminated magnetic anomaly responses (i.e., corresponding to min *Ω*) are not significantly affected by the noisy 15% of both noise, and they are significantly close to the true ones. Therefore, the BAO approach suggested here is stabilized to the different kind of noises that have been introduced. The RE of the recovered model parameters for both noise types is illustrated in Table 5.

**Table 5:** Numerical Model-2: True and recovered model parameters of the 15% noisy numerical example and the corresponding RE.

Model parameters	True value	Search range	Recovered value		RE (%)		*Ω*	
			15% RGN	15% AWGN	15% RGN	15% AWGN	15% RGN	15% AWGN
*K* (nT)	1500	500:2500	1480 ± 63.24	1460 ± 60.00	1.33	2.67	1.7*10^–7^	3.4*10^–5^
*z* (m)	7	1:10	7.2 ± 0.59	6.6 ± 0.60	2.86	5.71		
*x*_*o*_ (m)	0	–100:100	0 ± 1.83	0 ± 2.90	0.00	0.00		
*θ* (^o^)	– 65	–5:–90	– 68 ± 3.41	– 66 ± 3.42	4.62	1.54		

In order to test the stability of the suggested approach further with regard to the quantity of noises for the vertical dike model case, we have raised the proportion of noise to 20% for both the type of noises (RGN & AWGN) and reapplied the same procedure of the BAO approach to the noisy data anomalies. The recovered model parameters are displayed in Table 6 for the 20% noisy numerical examples for both types of noise (RGN and AWGN). The collected findings demonstrate that the recovered parameters still perform well when there is 20% noise for both types of noise (RGN and AWGN). Additionally, Table 6 shows the RE for each calculated model parameter for each type of noise in the event of a 20% noise quantity. Finally, it can be said that for the vertical dike model situation, the BAO technique proposed here is stable in terms of noise kinds and quantities.

**Table 6:** Numerical Model-2: True and recovered model parameters of the 20% noisy numerical example and the corresponding RE.

Model parameters	True value	Search range	Recovered value		RE (%)		*Ω*	
			20% RGN	20% AWGN	20% RGN	20% AWGN	20% RGN	20% AWGN
*K* (nT)	1500	500:2500	1580 ± 67.08	1400 ± 67.08	5.33	6.67	1.12*10^–5^	4.5*10^–5^
*z* (m)	7	1:10	6.4 ± 0.63	7.8 ± 0.59	8.57	11.43		
*x*_*o*_ (m)	0	– 100:100	4 ± 4.83	3 ± 5.11	0.00	0.00		
*θ* (^o^)	– 65	– 5:– 90	– 69 ± 3.35	– 70 ± 3.41	6.15	7.69		

### Numerical model-3

**Figure 9:**
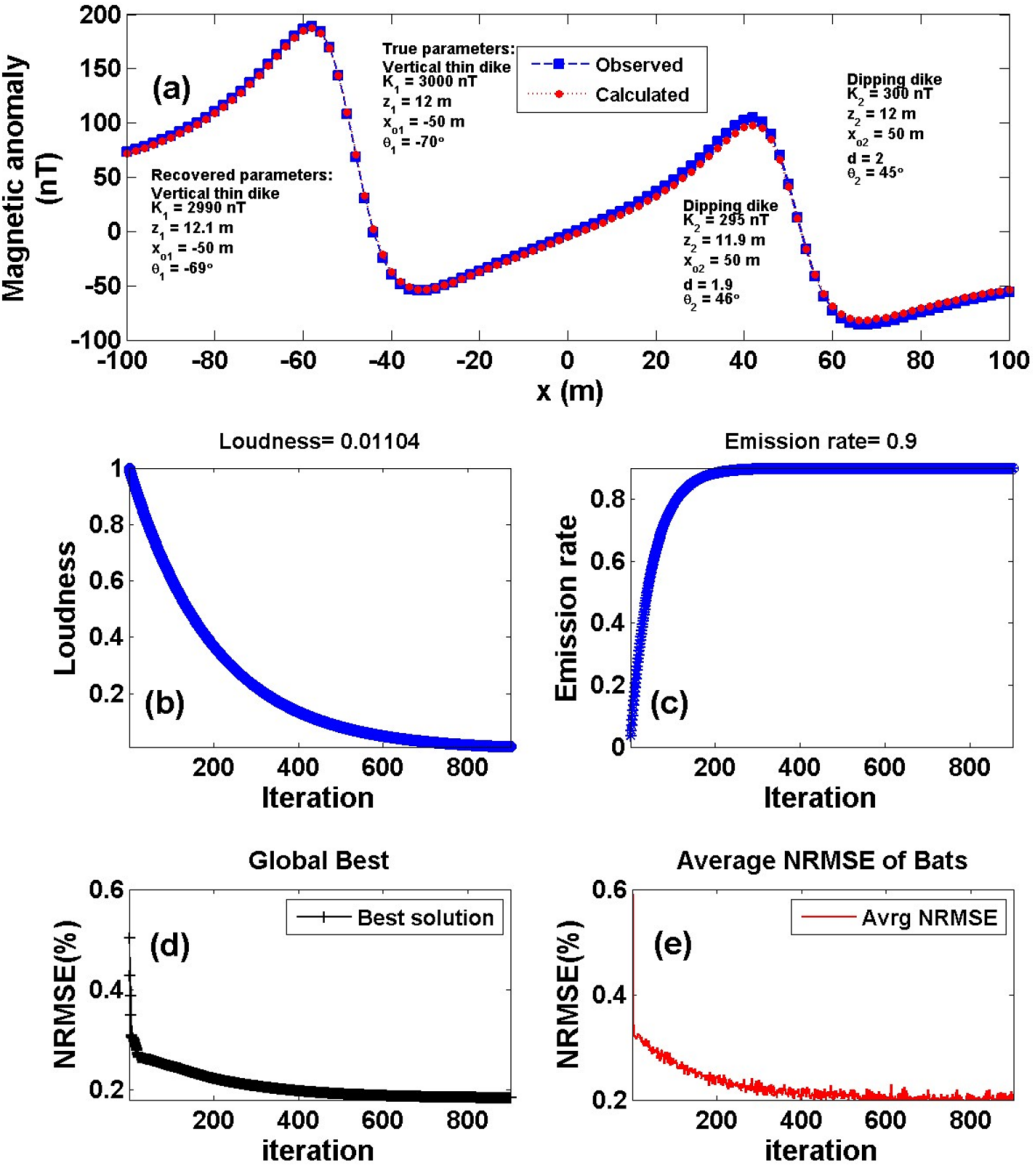
Numerical Model-3: Interference and multiple structure effect. (**a**) The composite magnetic anomaly generated by a vertical dike (or thin sheet) and 2D dipping dike model (True model parameters), as well as the calculated magnetic response of them (Recovered model parameters) using BAO approach, (**b**) loudness of the bats, (**c**) emission rat of the bats, (**d**) NRMSE of the global best solution (*Ω*) of the bats versus the iteration numbers, and (**e**) the average NRMSE of all the bats.

Interference affects the targets due to nearby multiple structures and also has an impact on the observed magnetic data. Therefore, we calculated the composite magnetic response (using Eqs. 7 and 8) for two neighboring source structures, called vertical dike (or thin sheet) model with true model parameters [*K*_*1*_ = 3000 nT, *z*_*1*_ = 12 m, *x*_*o1*_ =—50 m, *θ*_*1*_ =  70°] and a 2D dipping dike model with true model parameters [*K*_*2*_ = 300 nT, *z*_*2*_ = 12 m, *x*_*o2*_ = 50 m, *θ*_2_ = 45° and *d* = 2 m], with a profile length of 201 m (Fig. 9a) to check the impact of this multiple source structures on the accuracy of the recovered model parameters inferred using the BAO approach. Applying the aforementioned procedure of the BAO approach mentioned before, the measured magnetic anomaly of the two neighboring models is given in Fig. 9a. The obtained average loudness and emission rate of the bat of the composite anomaly are shown in Fig. 9b and c, respectively. The NRMSE of the global best solution (*Ω*) is shown in Fig. 9d, and the average NRMSE of all the bats is shown in Fig. 9e. Table 7 shows the recovered model parameters of the two interference source models are semi-identical to the true ones. Also, Table 7 shows the RE of the recovered model parameters for each source model. The result explains that the BAO approach can give accurate results in the case of the presence of multiple sources.

**Table 7:** Numerical Model-3: True and recovered model parameters of the composite interference multiple structures and the corresponding RE.

Model parameters	True value		Search range	Recovered value		RE (%)		*Ω*
	Vertical dike	Dipping dike		Vertical dike	Dipping dike	Vertical dike	Dipping dike	
*K* (nT)	3000	300	100:5000	2990 ± 7.07	295 ± 4.08	0.33	1.67	2.6 * 10^–3^
*z* (m)	12	12	1:20	12.1 ± 0.08	11.9 ± 0.08	0.83	0.83	
*x*_*o*_ (m)	– 50	50	–100:100	– 50 ± 0.00	50 ± 0.00	0.00	0.00	
*θ*	– 70	45	–90:90	– 69 ± 0.82	46 ± 0.81	1.43	2.22	
*d* (m)		2	1:5		1.9 ± 0.08		5.00	

**Figure 10:**
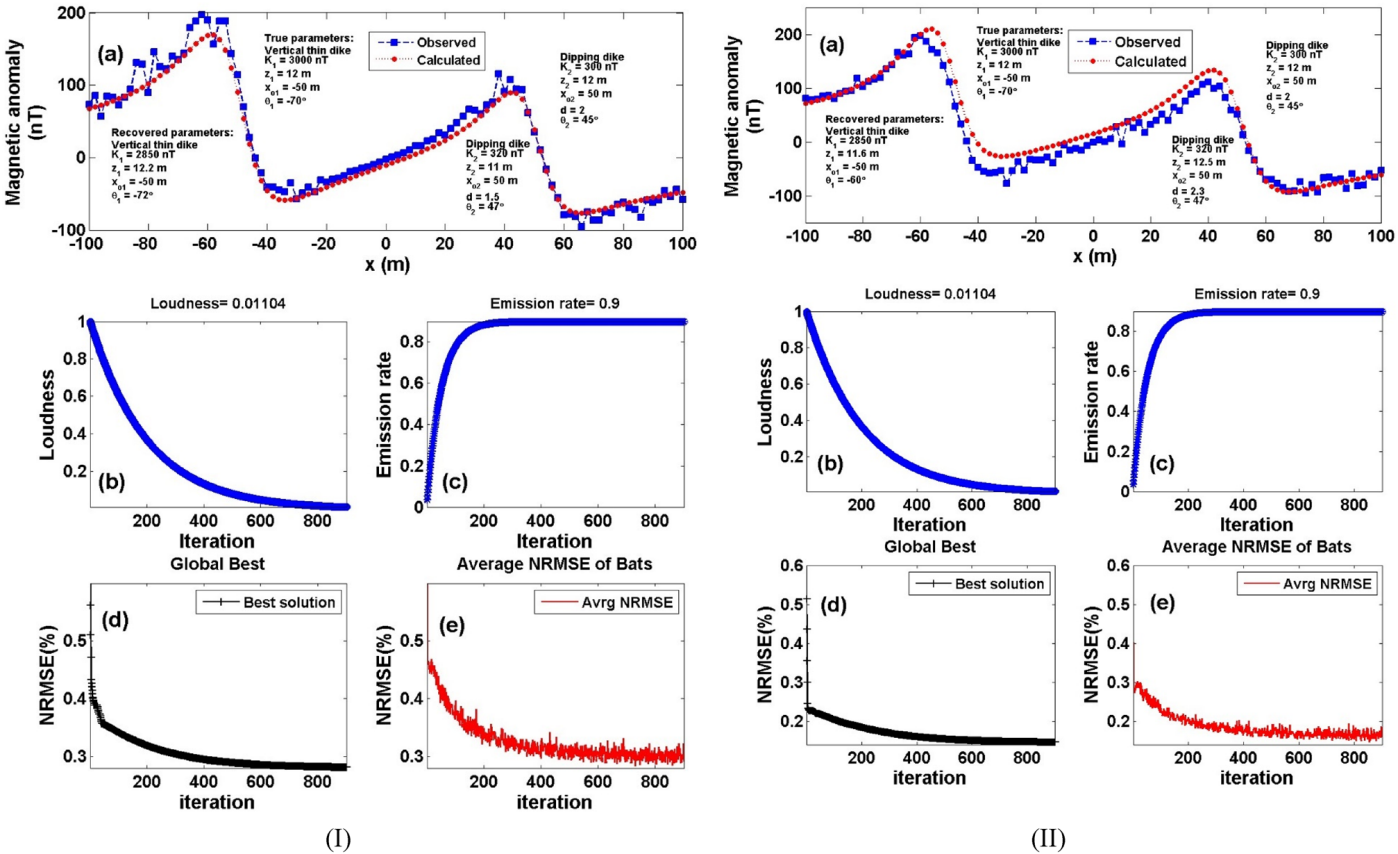
Numerical Model-3: Noisy interference and multiple structure effect (Fig. 9a) after contaminated with 10% RGN (I), and 10% AWGN (II). (**a**) The noisy composite magnetic anomaly generated by the vertical dike (or thin sheet) and 2D dipping dike model (True model parameters), as well as the calculated magnetic response of them (Recovered model parameters) using BAO approach, (**b**) loudness of the bats, (**c**) emission rat of the bats, (**d**) NRMSE of the global best solution (*Ω*) of the bats versus the iteration numbers, and (**e**) the average NRMSE of all the bats.

To test the stability of the proposed BAO approach on multiple and surrounding structure effects, we have contaminated the composite magnetic anomaly response of Fig. 9a with two distinct forms of noise that were previously described as RGN and AWGN with 10% noise amounts (Fig. 10a). By applying the BAO approach scheme to the noisy composite anomalies, the best obtained model parameter of the recovered models will be corresponding to the minimum NRMSE of the objective functions (*Ω*). Figure 10 shows the noisy contaminated composite magnetic anomaly of the two surrounded sources after adding the 10% RGN (I) and 10% AWGN (II) to the composite data shown in Fig. 9a, as well as the calculated magnetic response after obtaining the best model parameters using the BAO inversion approach (panel a). The loudness, emission rat, the NRMSE of the global best solution (*Ω*), and the average NRMSE of all the bats are shown in panels b, c, d, and e of Fig. 10, respectively.

Tables 8 and 9 show the results of the recovered model parameters of the noisy composite magnetic anomaly for both the distinct noise forms (RGN and AWGN). The recovered model parameters of the two polluted source models are still in good coincidence with the actual ones. In addition, Tables 8 and 9 illustrate the RE corresponding to each obtained model parameter for each distinct noise form. As a result, it's reasonable to conclude that the BAO technique provided here is stable in the presence of the distinct noise forms that are being intruded into the modeled data.

**Table 8:** Numerical Model-3: True and recovered model parameters of the noisy composite interference multiple structures after added 10% RGN to the composite anomaly, and the corresponding RE.

Model parameters	True value		Search range	Recovered value		RE (%)		*Ω*
	Vertical dike	Dipping dike		Vertical dike	Dipping dike	Vertical dike	Dipping dike	
*K* (nT)	3000	300	100:5000	2850 ± 129	320 ± 42.42	5.00	6.67	1.5 * 10^–8^
*z* (m)	12	12	1:20	12.2 ± 0.56	11 ± 0.60	1.67	8.33	
*x*_*o*_ (m)	– 50	50	– 100:100	– 50 ± 0.97	50 ± 0.97	0.00	0.00	
*θ*	– 70	45	– 90:90	– 72 ± 5.99	47 ± 6.00	2.86	4.44	
*d* (m)		2	1:5		1.5 ± 0.43		25.00

**Table 9:** Numerical Model-3: True and recovered model parameters of the noisy composite interference multiple structures after added 10% AWGN to the composite anomaly, and the corresponding RE.

Model parameters	True value		Search range	Recovered value		RE (%)		*Ω*
	Vertical dike	Dipping dike		Vertical dike	Dipping dike	Vertical dike	Dipping dike	
*K* (nT)	3000	300	100:5000	2850 ± 129	320 ± 42.42	5.00	6.67	6.0 * 10^–9^
*z* (m)	12	12	1:20	11.6 ± 0.17	12.5 ± 0.59	3.33	4.17	
*x*_*o*_ (m)	– 50	50	–100:100	– 50 ± 0.97	50 ± 0.97	0.00	0.00	
*θ*	– 70	45	– 90:90	– 60 ± 6.02	47 ± 5.99	14.29	4.44	
*d* (m)		2	1:5		2.3 ± 0.34		15.00	

Based on the numerical dataset examples presented above, it can be inferred that the BAO approach proposed here is accurate, stable, and appropriate for the interpretation of real magnetic data, as explained in the next section.

### Examples of real cases

The applicability of the proposed BAO technique to real magnetic data was investigated in the next section using three published field cases from China and Egypt for iron ore deposits and metavolcanic basalt rock. The first case study example has been applied to the Galinge magnetic anomaly in China of an iron ore deposit in the Northwest Province. The second case example is the Weigang magnetic anomaly for iron ore deposit in the Eastern Province of China. Finally, third case study example from Egypt is the Hamrawein magnetic anomaly for metavolcanic basalt rock investigation.

For real cases examples the optimal tuning parameters are set as (Q = [0, 5], L = 1.0 and r = 0.9) that achieve the minimum NRMSE of the objective function and give a fast convergence to the optimum solution. The initial velocity (*V*_*i*_) at position (*X*_*i*_) was set to zero at the beginning of the inversion process. The search range is adopted to simulate more realistic cases where a priori information is absent. Therefore, the search space is chosen in both synthetic and real datasets based on the minimum NRMSE of the objective function (*Ω*) where the search range for the model parameters that will give the minimum *Ω* will be selected as the suitable search range.

### Case-1: Galinge magnetic anomaly, Northwest, China

**Figure 11:**
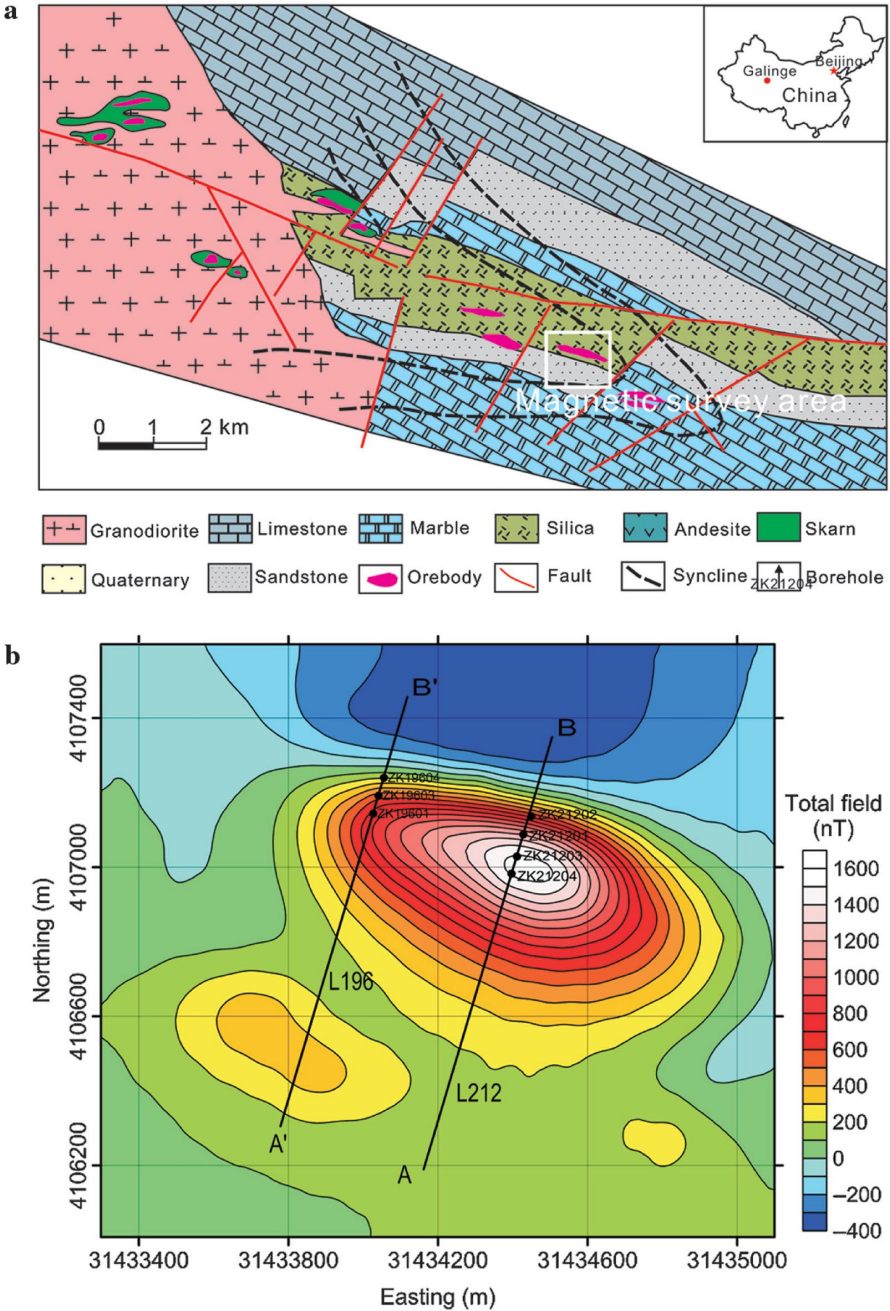
(**a**) The geological map of the Galinge area and (**b**) the total magnetic anomaly map of the Galinge iron ore deposit, northwestern of China^81^.

The Galinge iron ore deposit, located in the Qinghai region of northwest China, is one of the major skarn iron resources. The bedrock in the deposit is covered by broad and thick Quaternary (Q) gravel with thicknesses ranging from 117 to 210 m. The iron formation is mostly determined by the strata sequence, syngenetic breccia, volcanic, and subvolcanic rocks. The deposits' orebodies are mostly found in the lithological segments of the Ordovician Tanjianshan group (Fig. 11a). Skarnization and serpentinization are two significant wall-rock modifications that are strongly linked to mineralization. The principal ore minerals are magnetite and hematite, with minor quantities of siderite and hematite, and the average Fe grade is 37.16 percent. The Galinge iron ore deposit may have originated as a reformed and overlaid deposit as a result of volcanic emission and sedimentation^72–74^. The Quaternary gravel entirely covers the iron orebodies in this deposit. One of the most successful ways to discover magnetite deposits is through magnetic surveying. The total magnetic anomaly map reveals strong and regular magnetic anomalies with lengths and widths reaching 1200 and 500 m, respectively, with an ellipsoid form and extending northwest-southeast, with amplitudes over 1600 nT (Fig. 11b).

**Figure 12:**
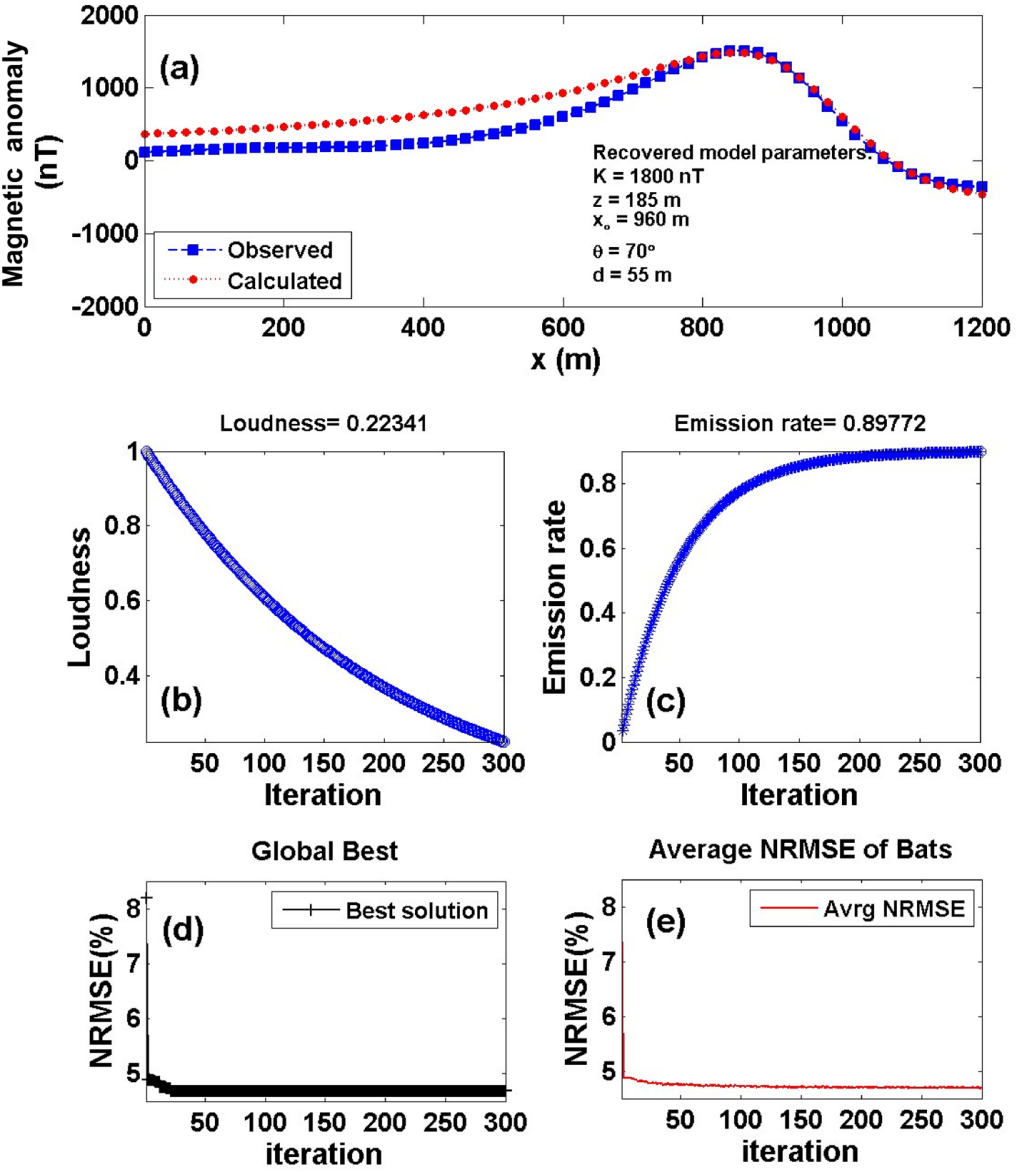
The Galinge magnetic anomaly, northwest, China. (**a**) The measured magnetic anomaly profile AB (L212) of Fig. 22 (blue squares), and the calculated best-fitting magnetic response (red circles) using BAO approach, (**b**) loudness of the bats, (**c**) emission rat of the bats, (**d**) NRMSE of the global best solution (*Ω*) of the bats versus the iteration numbers, and (**e**) the average NRMSE of all the bats.

**Figure 13:**
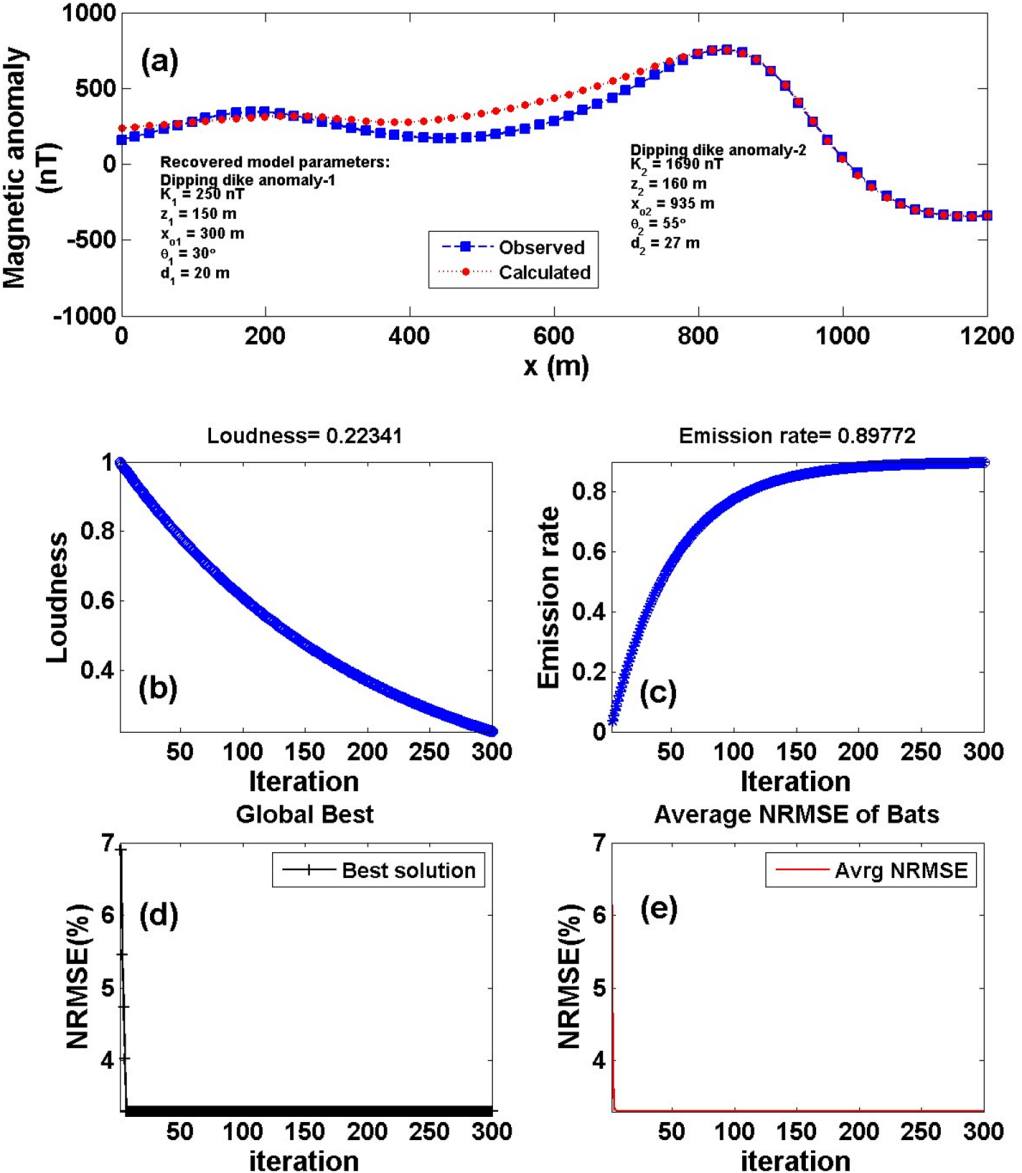
The Galinge magnetic anomaly, northwest, China. (**a**) The measured magnetic anomaly profile A'B' (L196) of Fig. 11 (blue squares), and the calculated best-fitting magnetic response (red circles) using BAO approach, (**b**) loudness of the bats, (**c**) emission rat of the bats, (**d**) NRMSE of the global best solution (*Ω*) of the bats versus the iteration numbers, and (**e**) the average NRMSE of all the bats.

Over the Galinge magnetic anomaly map, we have two profiles AB (L212) and A'B' (L196) in the northeast direction (Fig. 11b) were subjected to interpretation using the BAO inversion approach. The profiles AB and A'B' are digitized using a sampling interval of 20 m with a total profile length of 1200 m and are appeared in Figs. 12a and 13a, respectively. Applying the procedure of the BAO approach scheme on the Galinge magnetic anomaly of both profiles AB and A'B', the characteristics source parameters of the two anomalies can be estimated. Figures 12b,c and 13b,c show the average loudness and bat emission rate over the magnetic anomaly, respectively. As well, Figs. 12d,e and 13d,e show the NRMSE of the global best solution (*Ω*) and the average NRMSE of all the bats, respectively, of both anomalous profiles AB and A'B'.

**Figure 14:**
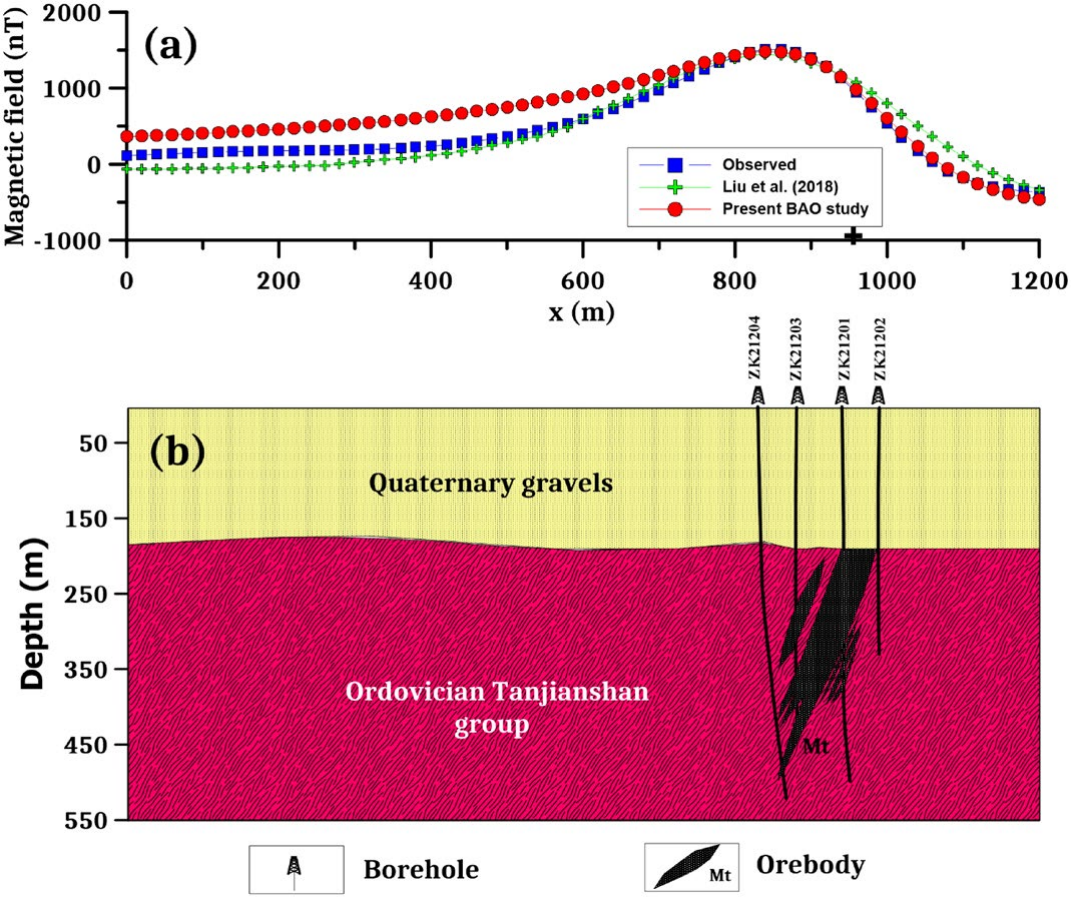
(**a**) Galinge magnetic profile AB and the interpreted calculated response using the present study BAO and other published technique (i.e. Liu et. al. 2018). (**b**) Drilling cross-section showing Galinge iron ore deposits (after^75^). The plus sign indicates the source position.

**Figure 15:**
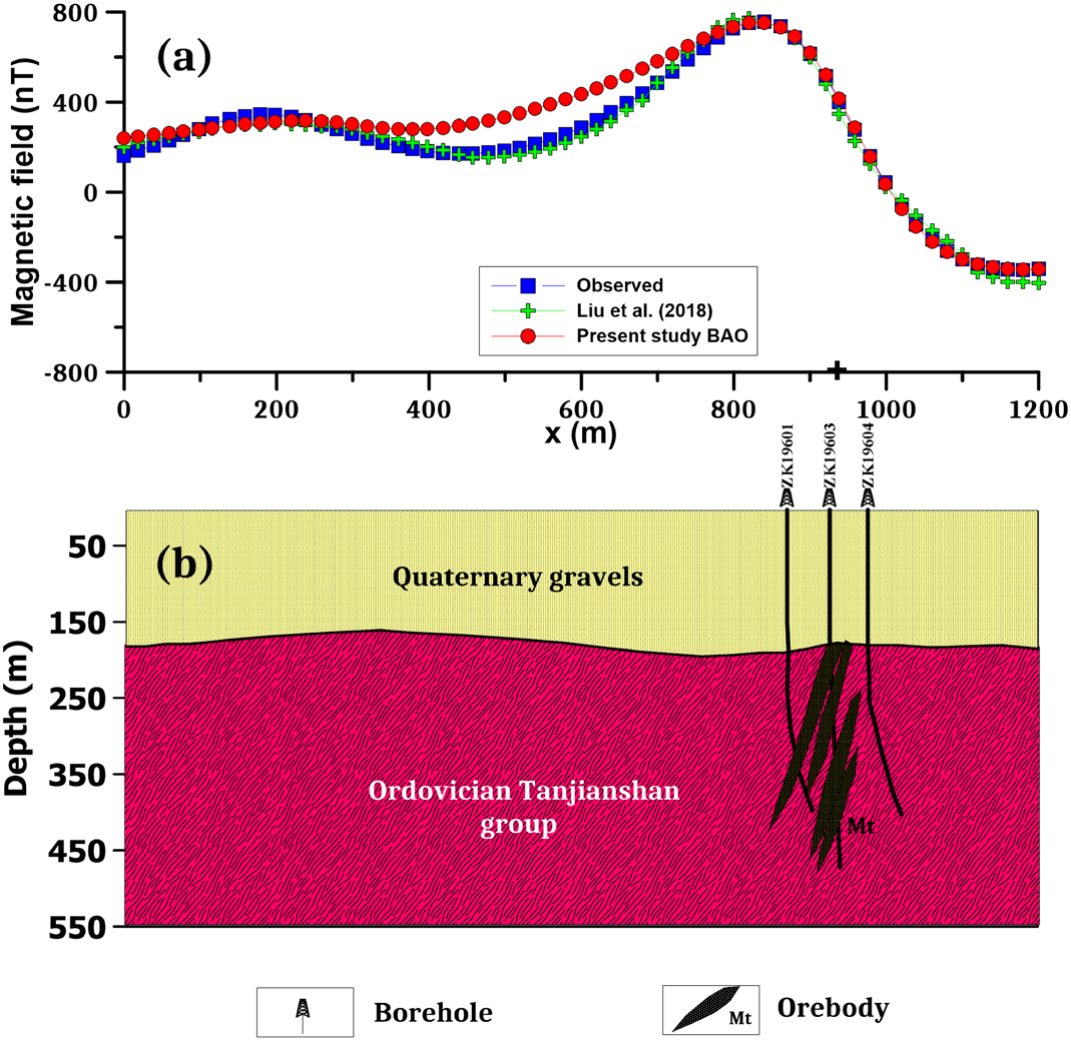
(**a**) Galinge magnetic profile A'B' and the interpreted calculated response using the present study BAO and other published technique (i.e. Liu et. al. 2018). (**b**) Drilling cross-section showing Galinge iron ore deposits (after^75^). The plus sign indicates the source position.

The best interpreted model parameters are corresponding to the minimum objective function (*Ω*). For anomaly profile AB (L212), the minimum value is 4.57 and the best recovered model parameters are [*K* = 1800 ± 408 nT, *z* = 185 ± 2.5 m, *x*_*o*_ = 960 ± 7.89 m, *θ* = 70° ± 5.00, and d = 55 ± 4.08 m], which suggests that the effect of the profile AB of Galinge anomaly has resulted from 2D dipping dike-like structure. The observed and calculated magnetic anomalies are in good matching as shown in Fig. 12a as well as the obtained depth to the top is excellent compared with the depth inferred by drilling as shown in Fig. 14. For anomaly profile A'B' (L196), there are two distinct anomalies appear along the profile, the min *Ω* = 3.31 and the best recovered model parameters for the first anomaly are [*K*_*1*_ = 250 ± 25.00 nT, *z*_*1*_ = 150 ± 5.00 m, *x*_*o1*_ = 300 ± 3.75 m, *θ*_*1*_ = 30° ± 2.49 and d_1_ = 20 ± 0.50 m], and for the second anomaly are [*K*_*2*_ = 1690 ± 5.00 nT, *z*_*2*_ = 160 ± 5.01 m, *x*_*o2*_ = 935 ± 3.76 m, *θ*_*2*_ = 55° ± 2.50 and d_2_ = 27 ± 0.51 m], the results suggest that the effect of the profile A'B' of Galinge anomaly is due to two dipping dikes-like structure. The observed and calculated magnetic anomalies of the A'B' profile are well coincident as shown in Fig. 13a. In addition to the obtained depth to the top of the second anomaly is approved with drilling and is in excellent agreement with the depth to the top inferred by drilling as illustrated in Fig. 15.

The Galinge magnetic anomaly profiles AB and A'B' were interpreted by Liu et al. (2018) using the standard PSO inversion method with a depth ranging from 200 to 500 m for Profile AB and a depth ranging from 200 to 400 m for profile A'B'. The present study of the BAO approach interpreted the Galinge anomaly profiles AB and A'B' approximated by 2D dipping and vertical dikes with depth to the top of the ore deposits of 185 m and 160 m for the profiles AB and A'B', respectively, which agree very well with the drilling information of the four boreholes ZK21204, ZK21203, ZK21201, and ZK21202 for the Galinge anomaly profile AB and the three boreholes ZK19601, ZK19603, and ZK19604 of the Galinge anomaly profile A'B' (Fig. 14b and Fig. 15b), respectively. In addition, the BAO approach has a good matching compared to the other published technique^75^ (Fig. 14a and  15a).

### Case-2: Weigang magnetic anomaly, East, China

**Figure 16:**
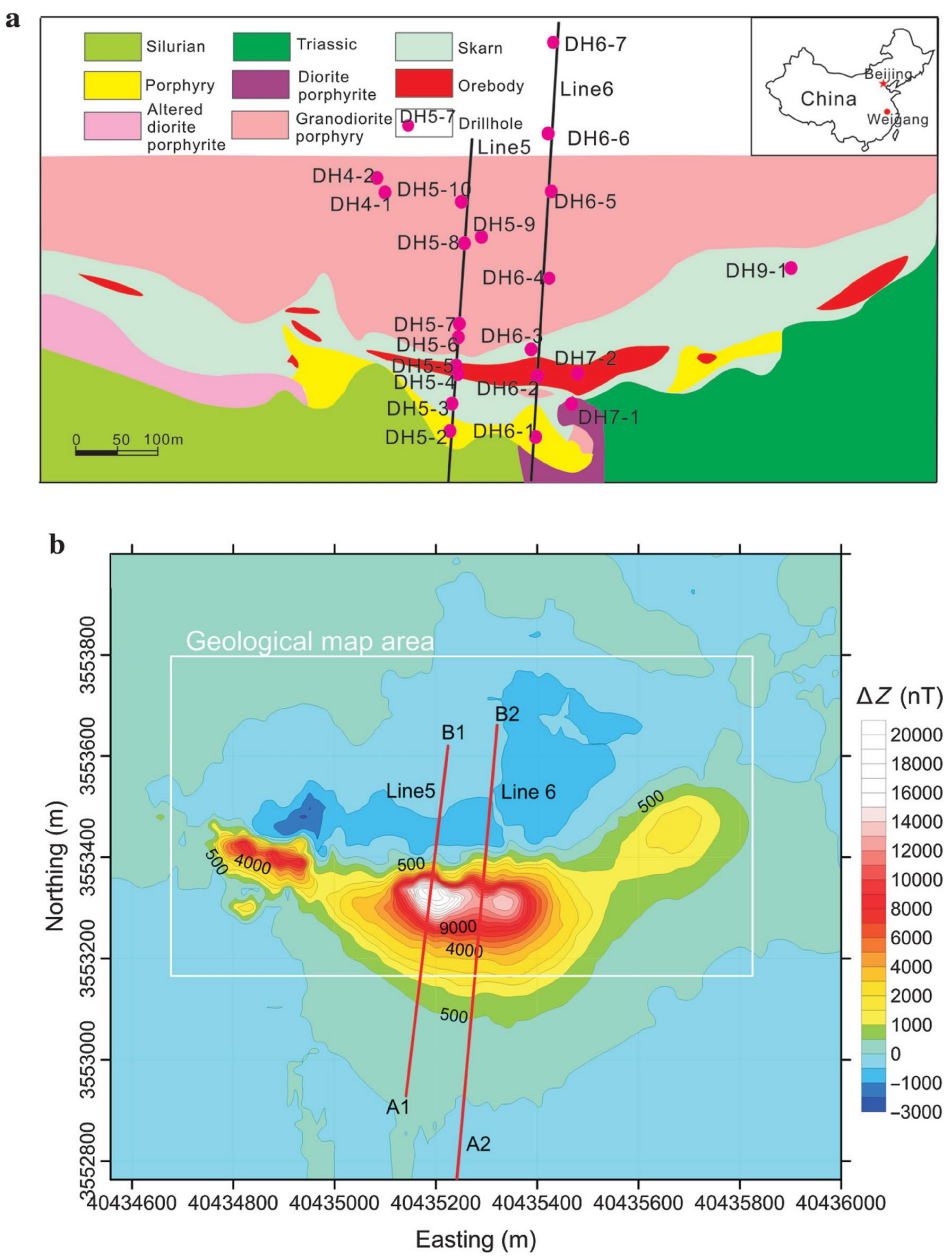
(**a**) The geological map of the Weigang area and (**b**) the vertical component of the magnetic anomaly (Δz) of the Weigang iron ore deposit, east, China^76^.

The Weigang iron ore deposits are discovered in the East of China, between the middle and lower sections of the Yangtze River's iron-copper metallogenic region. Silurian sandstone, Devonian quartz sandstone, Triassic limestone, and Cretaceous sandstone shale are some of the layers that have been exposed in the mining region. Late Yanshanian granodiorite porphyry, diorite porphyritic, and quartz diorite porphyry are the most important intrusive rocks (Fig. 16a). Weigang iron ore is a skarn-type magnetite deposit with ore bodies found in skarn contact zones between granodiorite porphyry, marble, and hornstone, which are considered high-temperature hydrothermal deposits generated by multiple mineralizations^76^. The vertical component (∆Z) of the magnetic anomaly of the Weigang region is depicted in Fig. 16b as a colored contour map. The magnetic anomaly is east–west, with amplitudes ranging from 1100 to 21,000 nanoteslas. The anomaly is around 1100 and 400 m long and wide.

**Figure 17:**
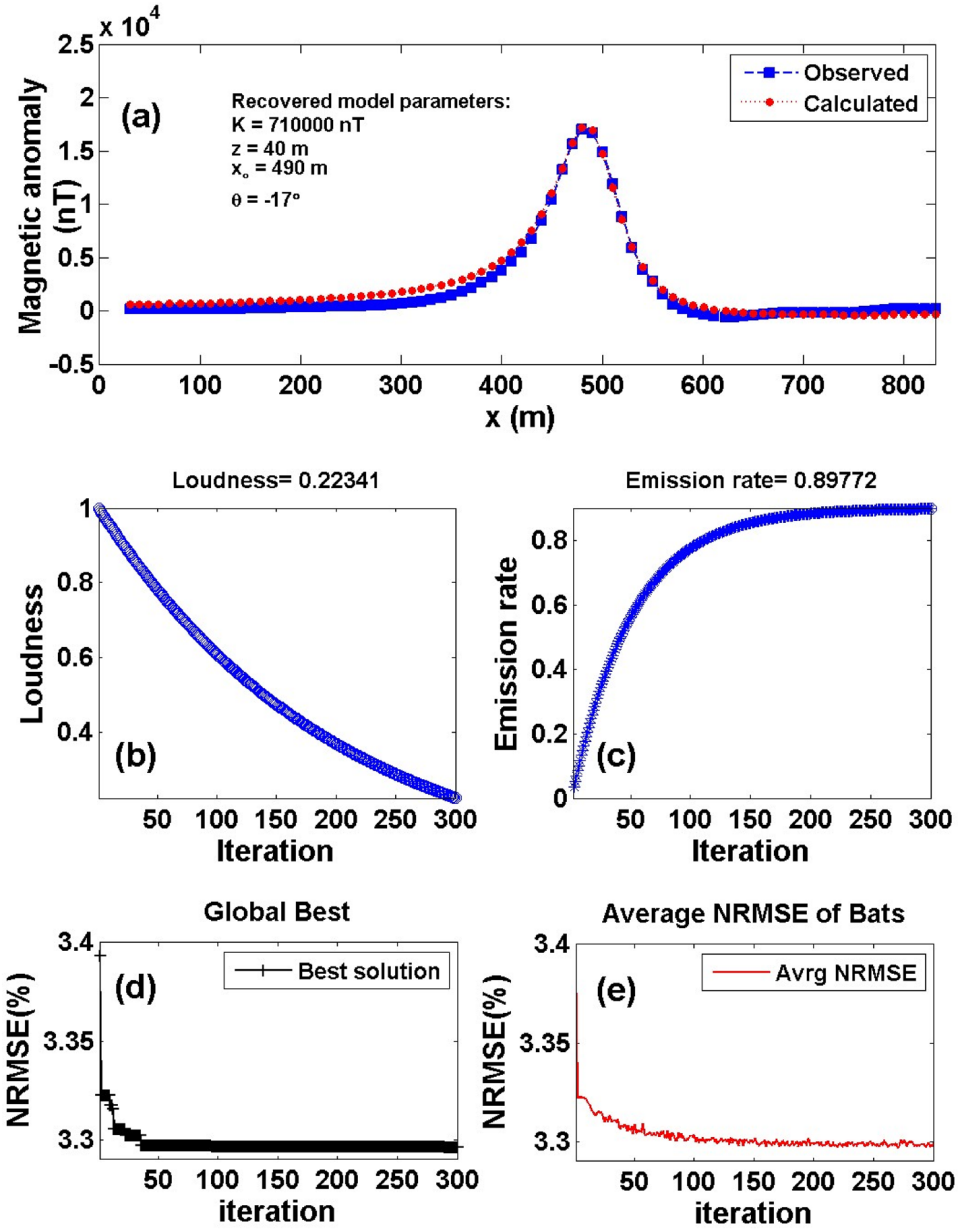
The Weigang magnetic anomaly, east, China. (**a**) The measured magnetic anomaly profile A1B1 (Line 5) of Fig. 16 (blue squares), and the calculated best-fitting magnetic response (red circles) using BAO approach, (**b**) loudness of the bats, (**c**) emission rat of the bats, (**d**) NRMSE of the global best solution (*Ω*) of the bats versus the iteration numbers, and (**e**) the average NRMSE of all the bats.

**Figure 18:**
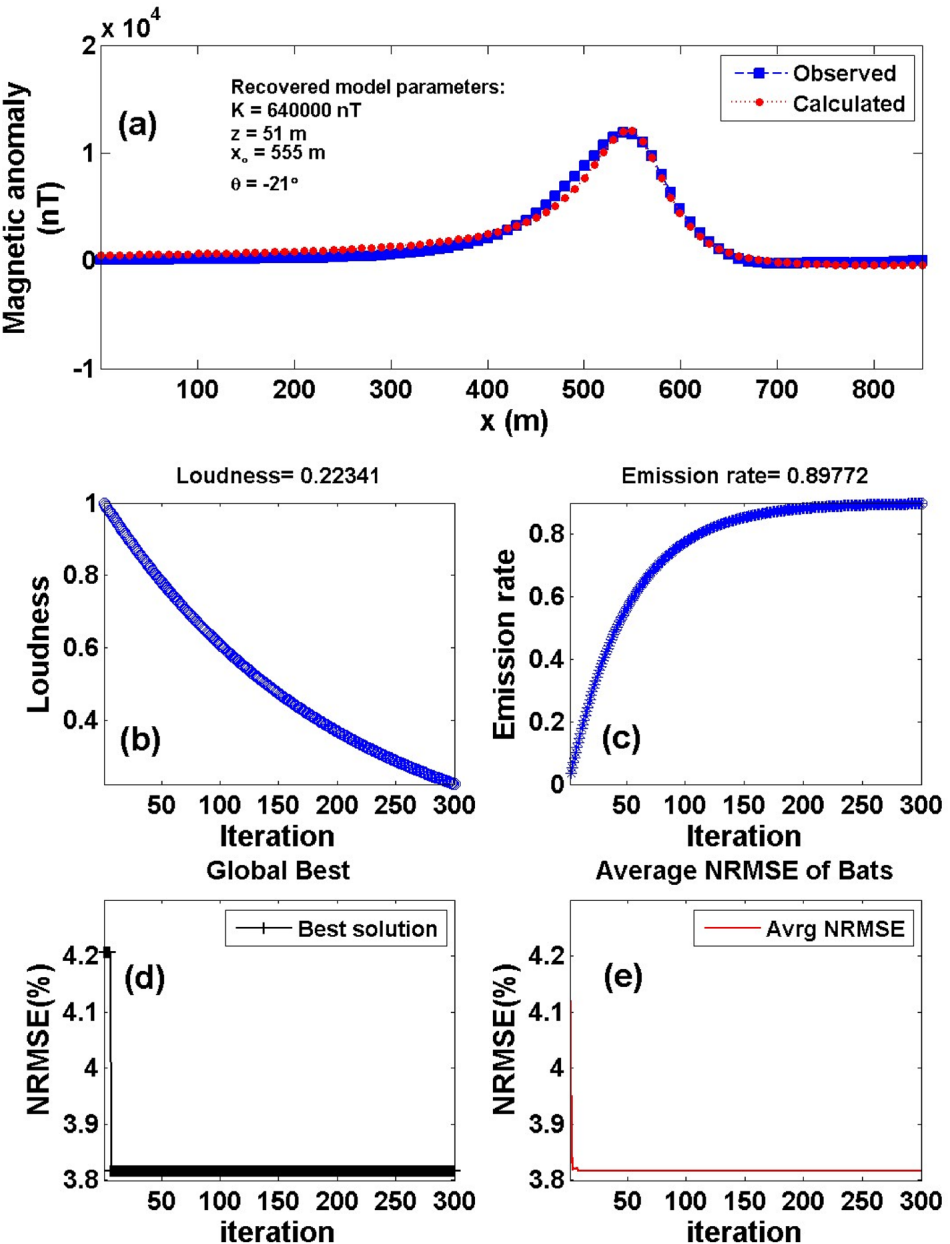
The Weigang magnetic anomaly, east, China. (**a**) The measured magnetic anomaly profile A2B2 (Line 6) of Fig. 16 (blue squares), and the calculated best-fitting magnetic response (red circles) using BAO approach, (**b**) loudness of the bats, (**c**) emission rat of the bats, (**d**) NRMSE of the global best solution (*Ω*) of the bats versus the iteration numbers, and (**e**) the average NRMSE of all the bats.

Two profiles are taken across the Weigang magnetic anomaly map, A1B1 (Line 5) and A2B2 (Line 6) in a roughly north–south direction (Fig. 16b) to investigate the iron ore-deposits in the region. The two profiles were subjected to interpretation using the BAO inversion approach. A 10-m sampling interval was used to digitize the two profiles A1B1 and A2B2 with the total length of 800 and 850 m as shown in Fig. 17a and Fig. 18a, respectively. Performing the BAO scheme on the Weigang magnetic anomaly of both profiles A1B1 and A2B2, the characteristics source parameters of the two anomalies can be obtained. Figures 17b,c and 18b,c show the average loudness and bat emission rate over the magnetic anomaly, respectively. In addition, Fig. 17d,e and Fig. 18d,e show the NRMSE of the global best solution (*Ω*), and the average NRMSE of all the bats, respectively, of both anomalous profiles A1B1 and A2B2.

**Figure 19:**
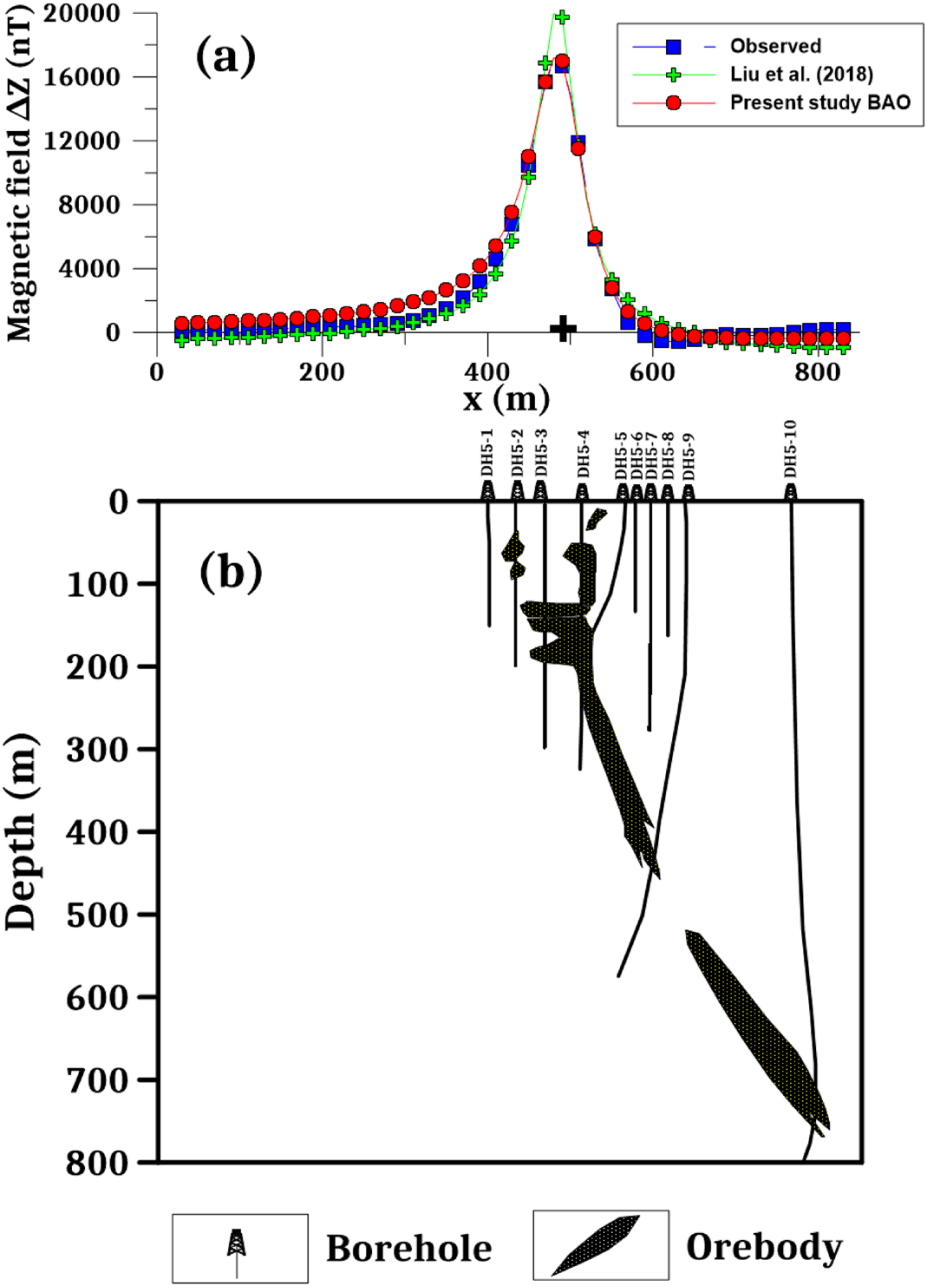
(**a**) Weigang magnetic profile A1B1 (Line 5) and the interpreted calculated response using the present study BAO and other published technique (i.e. Liu et. al. 2018). (**b**) Drilling cross-section showing Weigang iron ore deposits (after^75^). The plus sign indicates the source position.

**Figure 20:**
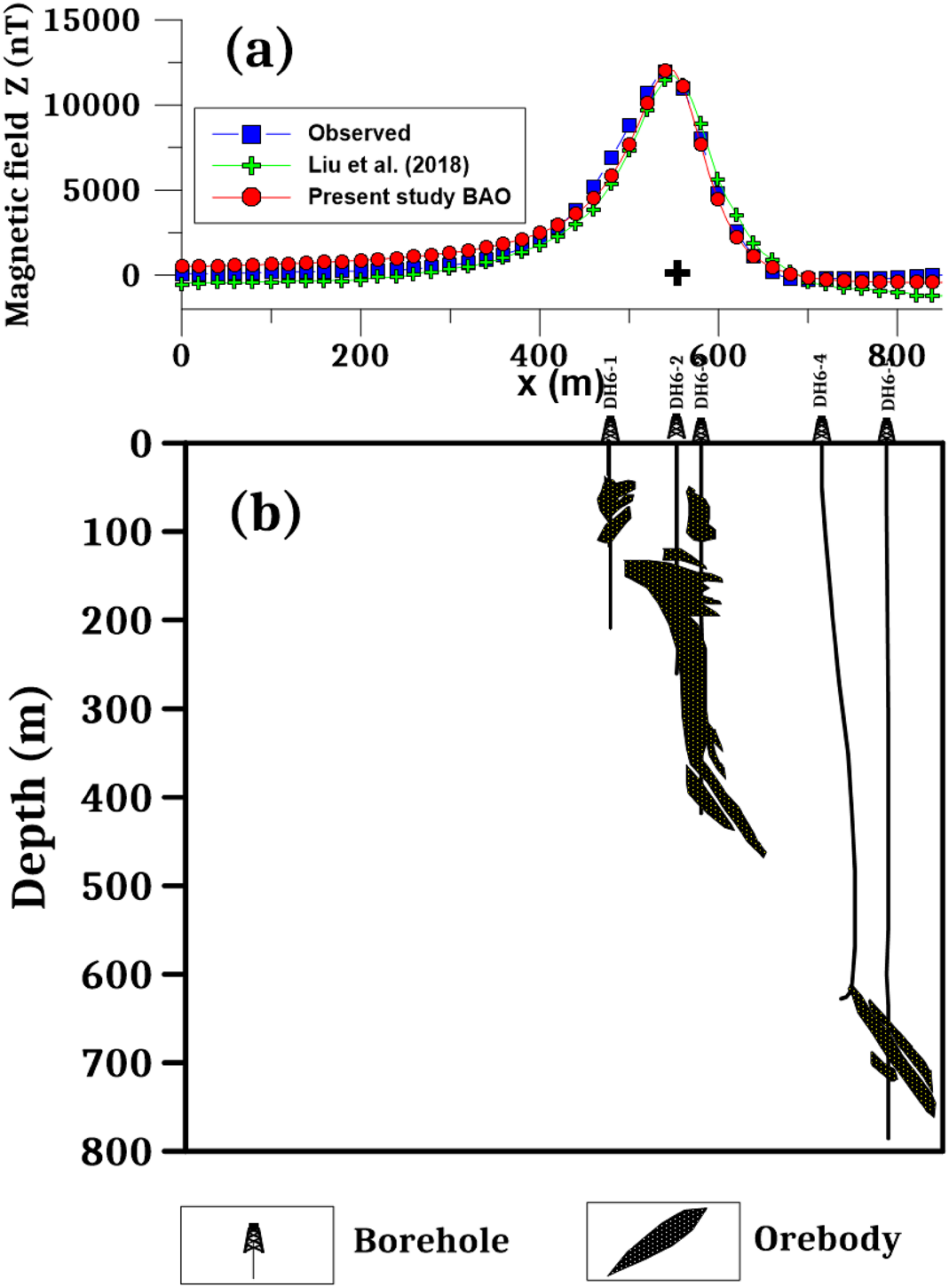
(**a**) Weigang magnetic profile A2B2 (Line 6) and the interpreted calculated response using the present study BAO and other published technique (i.e. Liu et. al. 2018). (**b**) Drilling cross-section showing Weigang iron ore deposits (after^75^). The plus sign indicates the source position.

The best recovered model parameters will be obtained when the optimal solution reaches the minimum of the objective function (*Ω*). For anomaly profile A1B1 (Line 5), the min *Ω* = 3.29 and the best recovered model parameters are [*K* = 710,000 ± 99.05 nT, *z* = 40 ± 2.00 m, *x*_*o*_ = 490 ± 1.24 m, and *θ* = -17° ± 2.00], which suggests that the effect of the profile A1B1 of the Weigang anomaly results from the vertical dike-like structure. The observed and calculated magnetic anomalies are in good matching as shown in Fig. 17a and the obtained depth to the top is excellent compared with the depth inferred by drilling as shown in Fig. 19. For anomaly profile A2B2 (Line 6), the min *Ω* = 3.81 and the best recovered model parameters are [*K* = 640,000 ± 115.56 nT, *z* = 51 ± 1.70 m, *x*_*o*_ = 550 ± 0.61 m, and *θ* = 21° ± 1.71]. The results suggest that the effect of the profile A2B2 of the Weigang anomaly is due to vertical dike-like structure. The observed and calculated magnetic anomalies of the A2B2 profile are well coincident as shown in Fig. 18a. Moreover, the obtained depth to the top of the second anomaly is approved with drilling and is in excellent agreement with the depth to the top inferred by drilling as illustrated in Fig. 20.

The Weigang magnetic anomaly profiles A1B1 and A2B2 have been interpreted also by Liu et al.^75^ using the standard PSO inversion method. The Weigang anomaly A1B1 profile (Line 5) was interpreted as a semi-vertical dike with a depth to the top of about 50 m and extended to 500 m. While the Weigang anomaly A2B2 profile (Line 6) was interpreted as a nearly vertical dike with a depth range from 100 to 400 m. The suggested BAO approach interpreted the Weigang anomaly profiles A1B1 and A2B2 approximated by vertical dikes with depth to the top of the ore deposits of 50 and 40 m for the profiles A1B1 and A2B2, respectively, which has good matching with the drilling information of the boreholes DH5-2, and DH5-4 for the Weigang anomaly profile A1B1 and the boreholes DH6-1, DH6-2, and DH6-3 of the Weigang anomaly profile A2B2 (Figs. 19b and 20b), respectively. In addition, the present BAO approach has a good matching compared to the other published technique^75^ (Figs. 19a and 20a) for both A1B1 and A2B2 profiles.

### Case-3: Hamrawein magnetic anomaly, Red-Sea, Egypt

**Figure 21:**
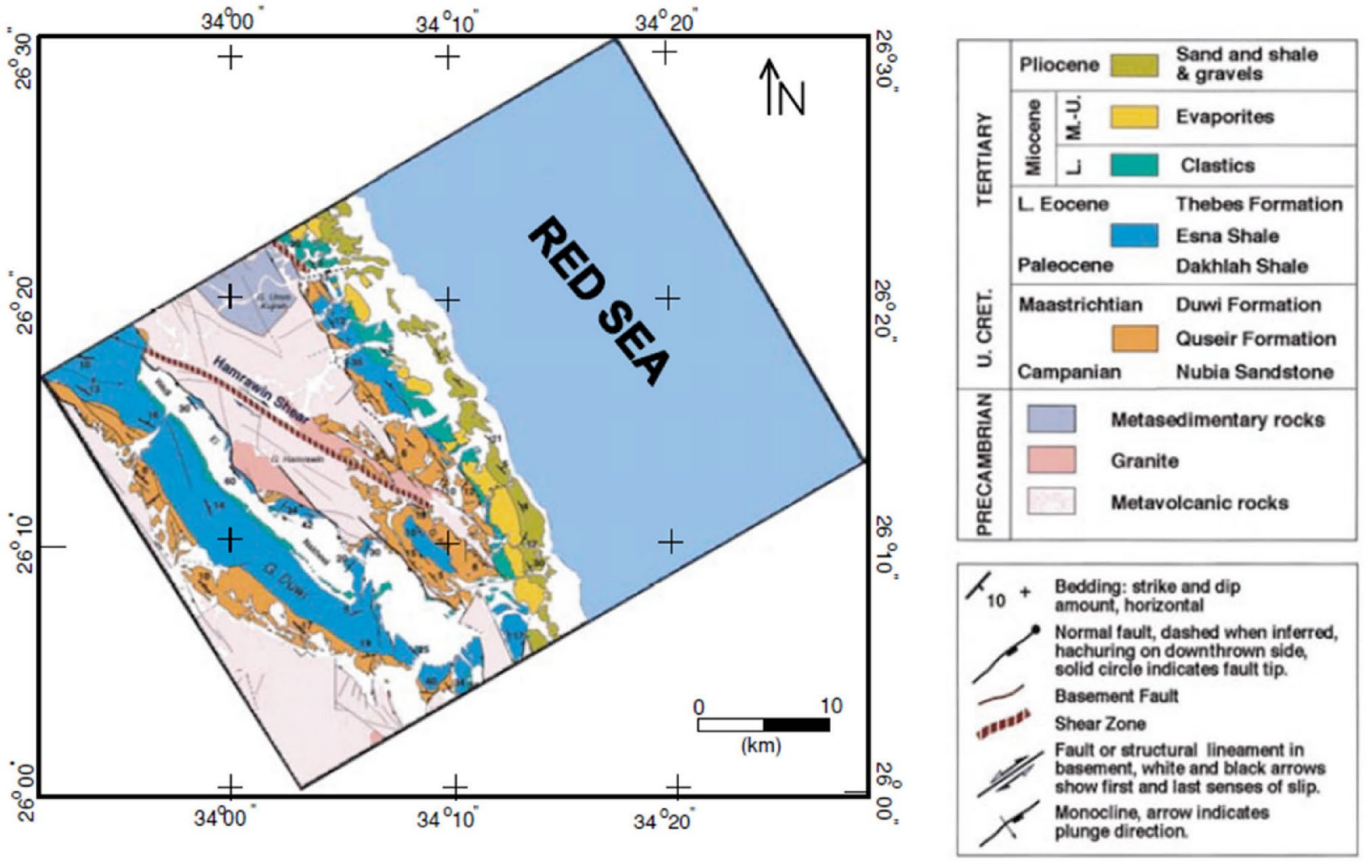
The Hamrawein’s geological map, Quseir area, Red-Sea, Egypt^79^.

**Figure 22:**
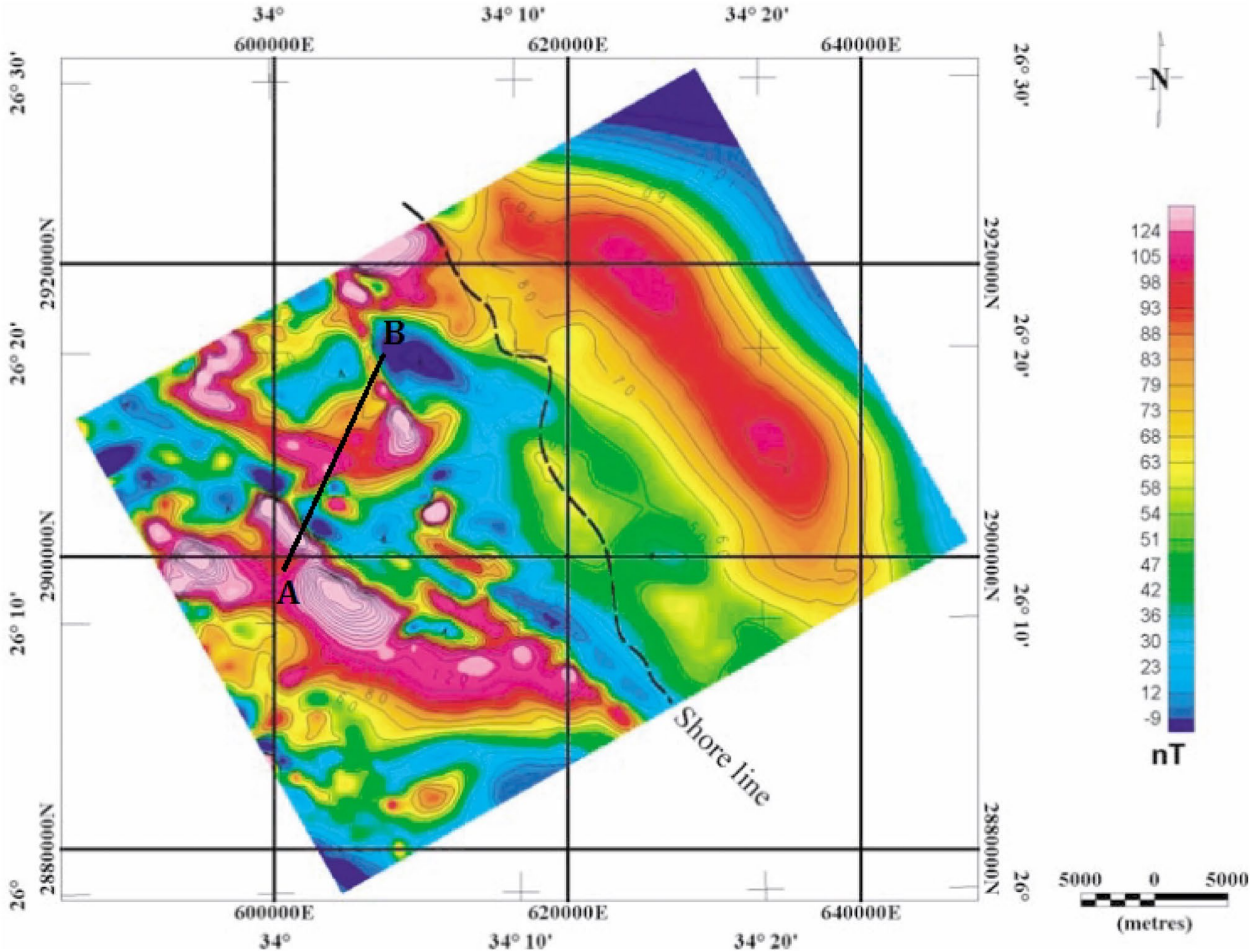
The Hamrawein’s total magnetic anomaly map, Red-Sea, Egypt^79^.

**Figure 23:**
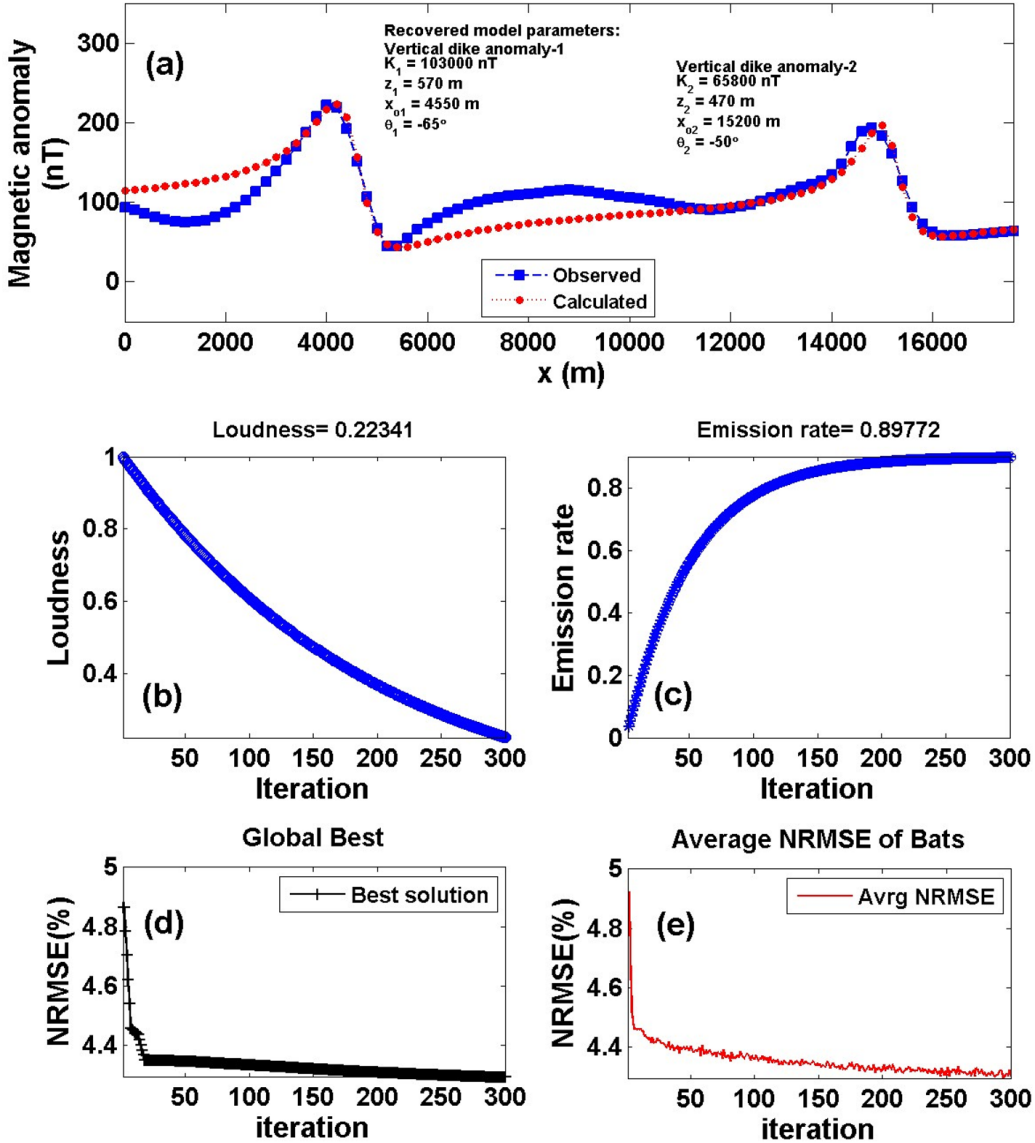
The Hamrawein magnetic anomaly, Red-Sea, Egypt. (**a**) The measured magnetic anomaly profile AB of Fig. 22 (blue squares), and the calculated best-fitting magnetic response (red circles) using BAO approach, (**b**) loudness of the bats, (**c**) emission rat of the bats, (**d**) NRMSE of the global best solution (*Ω*) of the bats versus the iteration numbers, and (**e**) the average NRMSE of all the bats.

A highly defined aerial magnetic survey over the Hamrawein region, near the western shore of the Red Sea, Egypt, measured the total magnetic anomaly of the Hamrawein field^77^. In general, sedimentary and meta-volcanic rocks cover the Hamrawein region (Fig. 21), and the detected magnetic anomaly is made up of two primary anomalies (63, 64). The magnetic anomaly's profile AB was taken in the northeast over the total intensity map of Hamrawein (Fig. 22) to investigate the metavolcanic basalt rocks using the BAO approach. The Hamrawein anomaly profile AB with a length of 17,800 m and is digitized at 200-m sampling intervals (Fig. 23a).

Following the procedures of the BAO approach, the loudness, emission rate, the NRMSE of the global best solution (*Ω*), and the average NRMSE of all the bats are shown in Fig. 23b–e, respectively. The best interpretive model parameters are corresponding to the min (*Ω*). The min (*Ω*) is 4.29 and the best recovered model parameters for the first anomaly are [*K*_*1*_ = 103,000 ± 1500.00 nT, *z*_*1*_ = 570 ± 0.99 m, *x*_*o1*_ = 4550 ± 35.41 m, and *θ*_*1*_ =  65° ± 10.00], and for the second anomaly are [*K*_*2*_ = 65,800 ± 100.00 nT, *z*_*2*_ = 470 ± 1.01 m, *x*_*o2*_ = 15,200 ± 35.50 m, and *θ*_*2*_ = -50° ± 5.00], the results recommended that the two distinct anomalies of the Hamrawein profile AB is due to presence two vertical dikes-like structure. The observed and calculated magnetic anomalies of the Hamrawein profile AB have outstanding matching (Fig. 23a).

**Figure 24:**
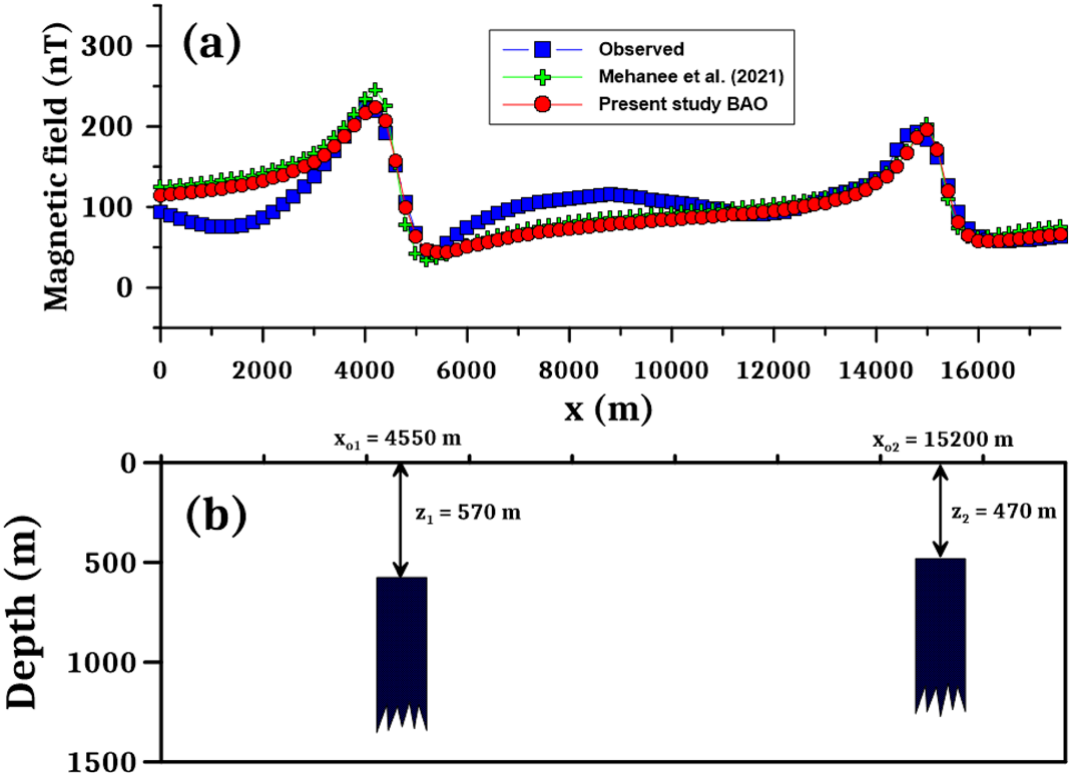
The Hamrawein magnetic anomaly, Red-Sea, Egypt. (**a**) The measured magnetic anomaly profile AB, and the calculated anomaly be using BAO approach compared with other published technique^19^. (**b**) The subsurface expected modeling using the present BAO approach.

Table 10 shows a comparison of the results achieved by the current method with those acquired by other published approaches^78,79^. Salem et al. (2005) interpreted the Hamrawein anomaly as two-sheet structures with depths of z_o1_ = 555.7 and z_o2_ = 441.2 m. According to Salem (2005), the depths are z_o1_ = 540 m and z_o2_ = 447 m. Salem^80^ used the local wavenumber (LW) approach with depths of z_o1_ = 432.6 m and z_o2_ = 422.8 m and the total gradient (TG) method with depths of z_o1_ = 486.5 m and z_o2_ = 440.4 m to explain the Hamrawein anomaly. Essa and Elhussein (2018) evaluate these anomalies by utilizing the particle swarm optimization (PSO) (z_o1_ = 623.05 m and z_o2_ = 494.14 m). Mehanee et al.^19^ interpreted the Hamrawein anomaly using the R-parameter technique and obtained the depths of the two-structures (z_o1_ = 480 m and z_o2_ = 440 m). We can be concluded that the depths obtained by the proposed technique (z_o1_ = 570 m and z_o2_ = 470 m) correspond well with those reported in the literature. In addition, Fig. 24 shows that the suggested BAO technique has a better matching than Mehanee et al.^19^ with observed data (Fig. 24a), as well as the subsurface expected modeling of the Hamrawein anomaly using the BAO approach (Fig. 24b).

**Table 10:** Case-3: Comparison results of the Hamrawein magnetic anomaly, Red-Sea, Egypt.

Model	Salem et al. (2005)		Salem (2005)		Salem (2011)		Present Study	
	First	Second	First	Second	First	Second	First	Second
Parameters	anomaly	anomaly	anomaly	anomaly	anomaly	anomaly	anomaly	anomaly
K (nT.m)	–	–	–	–	127,595.3	83,746.7	103,000 ± 1500	65,800 ± 100
*z* (m)	555.7	441.2	540	477	486.5	440.4	570 ± 0.99	470 ± 1.01
*x*_*o*_ (m)	4526.3	14,858	4530	14,850	–	–	4550 ± 35.41	15,200 ± 35.50
*θ* (^*o*^)	–	–	–	–	–	–	– 65 ± 10.00	50 ± 5.00
q	1.44	1.20	1.4	1.2	1.0	1.0	1.0	1.0

## Conclusions

In magnetic interpretation, determining the appropriate buried model for describing subsurface structures is critical. To analyze magnetic data, a global Bat algorithm optimization technique (BAO) was used to obtain the suitable model parameters (best model). After attaining the global best solution, the best-interpreted model parameters (amplitude coefficient, depth, source location, width, and index parameter angle) are executed. The BAO's designed inversion technique is simple, fast, accurate, and straightforward to apply to various magnetic datasets and does not require a priori information. Furthermore, it is capable effectively of handling the multi-models issue. Moreover, the efficiency and accuracy of the suggested method have been confirmed on numerical datasets with different types of noise (RGN and AWGN) and amounts (10%, 15% and 20%). Finally, the BAO approach is fruitfully utilized in three different real cases from China and Egypt for ore deposits exploration and metavolcanics rock investigations.

## Data Availability

The authors declare that the data is available upon request.
